# Targeted Enrichment of Engineered Bacteria on Triple‐Negative Breast Cancer Turns Immunologically Cold Tumors Hot

**DOI:** 10.1002/advs.202510171

**Published:** 2025-09-30

**Authors:** Xuanxiang Zhai, Xiaoyi Shi, Xiao Liu, Xiaotong Gu, Wenting Li, Xiangjun Chen, Wei Hong

**Affiliations:** ^1^ School of Pharmacy Shandong New Drug Loading & Release Technology and Preparation Engineering Laboratory Binzhou Medical University 346 Guanhai Road Yantai 264003 P. R. China

**Keywords:** engineered bacteria, in situ activation, mild photothermal immunotherapy, self‐blocking immune checkpoint, triple‐negative breast cancer

## Abstract

Tumor immunotherapy has garnered significant attention, however, several notable challenges remain to be addressed, including: 1) enhancing active targeting, 2) effectively shielding immune checkpoints, and 3) inducing the transformation of “cold” tumor into “hot”. In this study, an “all‐in‐one” extracellular anaerobic bacterial nanocomposite system is constructed. *Escherichia coli* Nissle 1917 (EcN) is enveloped with polydopamine via self‐polymerization (^PDA^EcN), subsequently conjugated with a chitosan oligosaccharide (COS) nanoparticle immunostimulant backpack, denoted as ^PDA^EcN/COS. The PDA coating is capable of concealing EcN polysaccharides, masking the immunogenic bacterial surface antigens, and inducing a mild photothermal therapy (PTT) effect. Moreover, ^PDA^EcN/COS exhibits targeted accumulation in hypoxic regions of solid tumors and demonstrates pronounced enrichment on tumor cell surfaces, attributed to the bacterial hypoxic region targeting ability and adhesive properties of PDA, physically obstructing the immune checkpoint. Simultaneously, both EcN and COS markedly enhanced the transformation of M2 macrophages into the M1 phenotype, whereas mild PTT‐induced immunogenic cell death (ICD) further mitigated the immunosuppressive nature of the hypoxic tumor microenvironments (TMEs). This integrated therapeutic approach eradicated tumors without eliciting metastasis or discernible side effects following a single injection and laser irradiation in a murine 4T1 cancer model. Ultimately, the immunostimulatory capacity of ^PDA^EcN/COS is considered to hold significant potential for developing novel and efficacious therapies for immunologically “cold” triple‐negative breast cancer (TNBC).

## Graphical abstract

In this study, an “all‐in‐one” engineered anaerobic bacterial system (^PDA^EcN/COS) is developed for the treatment of immunologically “cold” TNBC. This nanoplatform is capable of colonizing tumor cell surfaces and implementing “self‐blocking” of immune checkpoints, while simultaneously suppressing tumor cells' “don't eat me” signals and delivering “eat me” signals to immune cells. The system integrates four key therapeutic functions after automatically migrating toward hypoxic tumor regions: blocking immune checkpoints, inducing tumor ICDs, promoting tumor‐associated macrophage (TAM) polarization, and facilitating bacterial therapy.

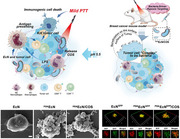

## 1 Introduction

Triple‐negative breast cancer (TNBC) is widely recognized as an exceptionally invasive and rapidly growing subtype, characterized by unfavorable outcomes and diminished patient survival rates.^[^ ^1^, ^2^ ^]^ Conventional treatments, including chemotherapy and surgical excision, encounter challenges such as multidrug resistance and recurrence, thereby resulting in limited therapeutic efficacy.^[^ ^3^, ^4^ ^]^ Subsequently, cancer immunotherapy emerged and has become a prominent focus in contemporary antitumor research. Immunotherapy represents a treatment approach designed to stimulate the body's complete immune response against malignant cells, thereby achieving therapeutic outcomes.^[^ ^5^, ^6^ ^]^ The clinically employed immunotherapies demonstrating favorable results predominantly encompass immune checkpoint blockade (ICB) therapy and chimeric antigen receptor (CAR) T‐cell therapy.^[^ ^7^, ^8^, ^9^, ^10^ ^]^ Nevertheless, ICB treatment tends to cause dermal adverse effects and autoimmunity complications, while CAR T cell therapy potentially triggers cytokine storm syndrome.^[^ ^11^, ^12^ ^]^

Immunogenic cell death (ICD) constitutes another strategy to activate antitumor immunity. Following induction treatment, tumor cells are capable of releasing tumor‐associated antigens and damage‐associated molecular patterns (DAMPs), which subsequently stimulate dendritic cells (DCs) and boost their antigen‐presenting abilities, thereby facilitating the mobilization and stimulation of cytotoxic T lymphocytes (CTLs) to suppress tumor progression.^[^ ^13^, ^14^ ^]^ Photothermal therapy (PTT), characterized by its advantages of localized intervention, non‐invasiveness, and controllable irradiation and temperature, has been recognized as a novel paradigm for precise cancer therapy.^[^ ^15^, ^16^, ^17^, ^18^ ^]^ Conventionally, to establish a sufficiently harsh microenvironment for effective tumor ablation, rigorous photothermal heating exceeding 50 °C is necessitated, which unfortunately results in collateral damage to healthy tissues. Nonetheless, when the photothermal energy is reduced, the therapeutic efficacy of PTT is markedly compromised, and the PTT‐induced abscopal effect remains insufficient to suppress residual tumor margins. Recent observations have revealed that mild PTT, maintaining a relatively lower temperature of ≈45 °C, has been employed as an adjunctive measure in tumor therapy rather than as a direct cytotoxic approach.^[^ ^19^, ^20^, ^21^ ^]^ Mild thermal stimulation has been reported to activate the immune system, including motivation immune cells, promotion tumor T cells infiltration, and reinforcement innate and adaptive immune responses, which may increase the immunogenicity of tumors to reprogram the “cold” tumor microenvironments (TMEs).^[^ ^22^ ^]^

Furthermore, tumors employ diverse immune evasion strategies to circumvent immune surveillance and destruction. Thus, remodeling the tumor immune microenvironment is considered an effective means of enhancing antitumor immunity. Tumor‐associated macrophages (TAMs) represent the principal immunosuppressive cell population within tumors and are intimately linked to tumor invasion and metastasis.^[^ ^23^, ^24^ ^]^ Generally, macrophages are categorized into M1‐like macrophages, which exert antitumor activity, and M2‐like macrophages, which facilitate tumor proliferation. M1 and M2 macrophages possess the capacity for mutual conversion through the activation of related signaling pathways. Inducing the transition of M2 macrophages toward M1 phenotype can markedly diminish the quantity of M2 macrophages, augment the release of tumor necrosis factor‐alpha (TNF‐α), interleukin‐6 (IL‐6), and other cytokines, stimulate the antitumor immune response, and promote the eradication of tumor cells.^[^ ^25^, ^26^ ^]^ Numerous studies have confirmed that drug delivery systems utilizing this “turning enemies into friends” strategy possess significant efficacy in tumor treatment and offer promising prospects for therapeutic advancement.^[^ ^27^, ^28^, ^29^, ^30^ ^]^

Bacterial therapy has emerged as a novel modality for tumor treatment, capable of being employed either independently or synergistically with conventional therapeutic approaches.^[^ ^31^, ^32^, ^33^ ^]^ Extensive research examining the link between bacterial entities and cancer treatment demonstrated that bacterial organisms possessed the ability to suppress tumor development and spread by restricting tumor blood vessel formation and stimulating the immune responses of the host.^[^ ^34^, ^35^, ^36^ ^]^ In addition to their direct therapeutic role, bacteria possess an inherent ability to target tumor tissues owing to the hypoxic characteristics of the TMEs. The hypoxic necrotic regions within tumors furnish anaerobic and facultative anaerobic bacteria with essential nutrients and environmental conditions conducive to their colonization. Owing to this tumor‐targeting capability, bacteria have also been utilized as vectors for the delivery of therapeutic agents, ensuring precise targeting of tumors. To achieve this, researchers have predominantly employed electroporation, physical adsorption, and chemical grafting techniques to engineer nanoparticle‐loaded bacteria for targeted tumor therapy, resulting in favorable therapeutic outcomes.^[^ ^37^, ^38^, ^39^ ^]^

Chitosan oligosaccharide (COS) is the only naturally positively charged alkaline oligosaccharide, exhibiting significant potential in immunomodulation, anti‐tumor activity, and inflammation regulation. The anti‐inflammatory and immune‐enhancing properties of COS primarily depend on Toll‐like receptor 4, mannose receptor, and complement receptor 3 located on the surfaces of macrophages.^[^ ^40^, ^41^, ^42^ ^]^ These receptors cooperate with IFN‐γ to activate the transcription factor NF‐κB, thereby enhancing the tumoricidal capacity of macrophages. Therefore, bacteria loaded with COS can further promote the polarization of TAMs toward the M1 phenotype, thereby amplifying overall immunobiological responses.

**Scheme 1:**
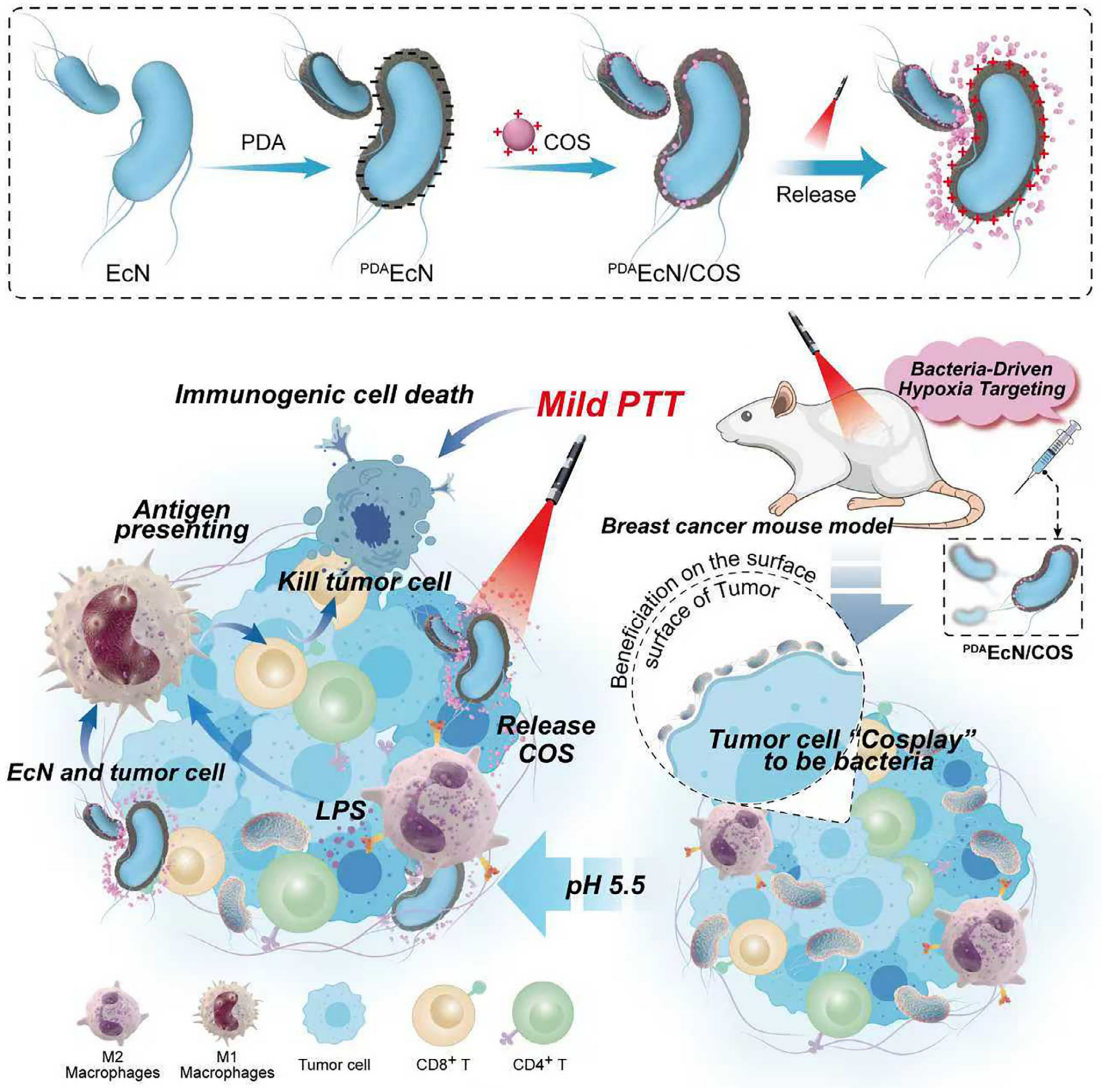
Schematic diagram of targeted enrichment of engineered bacteria in triple‐negative breast cancer turns immunologically cold tumors hot.

In this study, an “all‐in‐one” engineered anaerobic bacterial system (^PDA^EcN/COS) combining ICD, TAM polarization, and bacterial therapy was constructed. This system is based on polydopamine (PDA)‐coated *Escherichia coli* Nissle 1917 (^PDA^EcN) loaded with COS nanoparticles. TNBC (4T1) cells were selected as the model system. ^PDA^EcN/COS demonstrated autonomous migration toward hypoxic tumor regions, achieving the following outcomes: (I) Utilizing the extracellular characteristics of EcN alongside the adhesive properties of PDA, ^PDA^EcN/COS accumulated on tumor cell surfaces, effectively shielding immune checkpoints; (II) Macrophages were locally activated by EcN and COS, promoting the polarization of M2‐type TAMs toward the M1 phenotype; (III) PDA‐mediated mild PTT induced ICD in tumor cells, thereby promoting DC activation and augmenting CD8^+^ T cell‐mediated immune responses; (IV) M1‐type TAMs secreted TNF‐α and IL‐6, further activating T cells and amplifying overall immunobiological responses (Scheme 1). Distinct from traditional bacterial therapeutic strategies, ^PDA^EcN/COS was capable of colonizing tumor cell surfaces and “self‐blocking” immune checkpoints, simultaneously shielding tumor cells’ “don't eat me” signals and transmitting “eat me” signals to immune cells. This work provided a novel therapeutic concept for bacteria‐mediated and tumor‐targeted immunotherapy, offering an innovative avenue for the treatment of immunologically “cold” TNBC.

## 2 Results and Discussion

### 2.1 Preparation and Characterization of PDAEcN/COS

**Figure 1:**
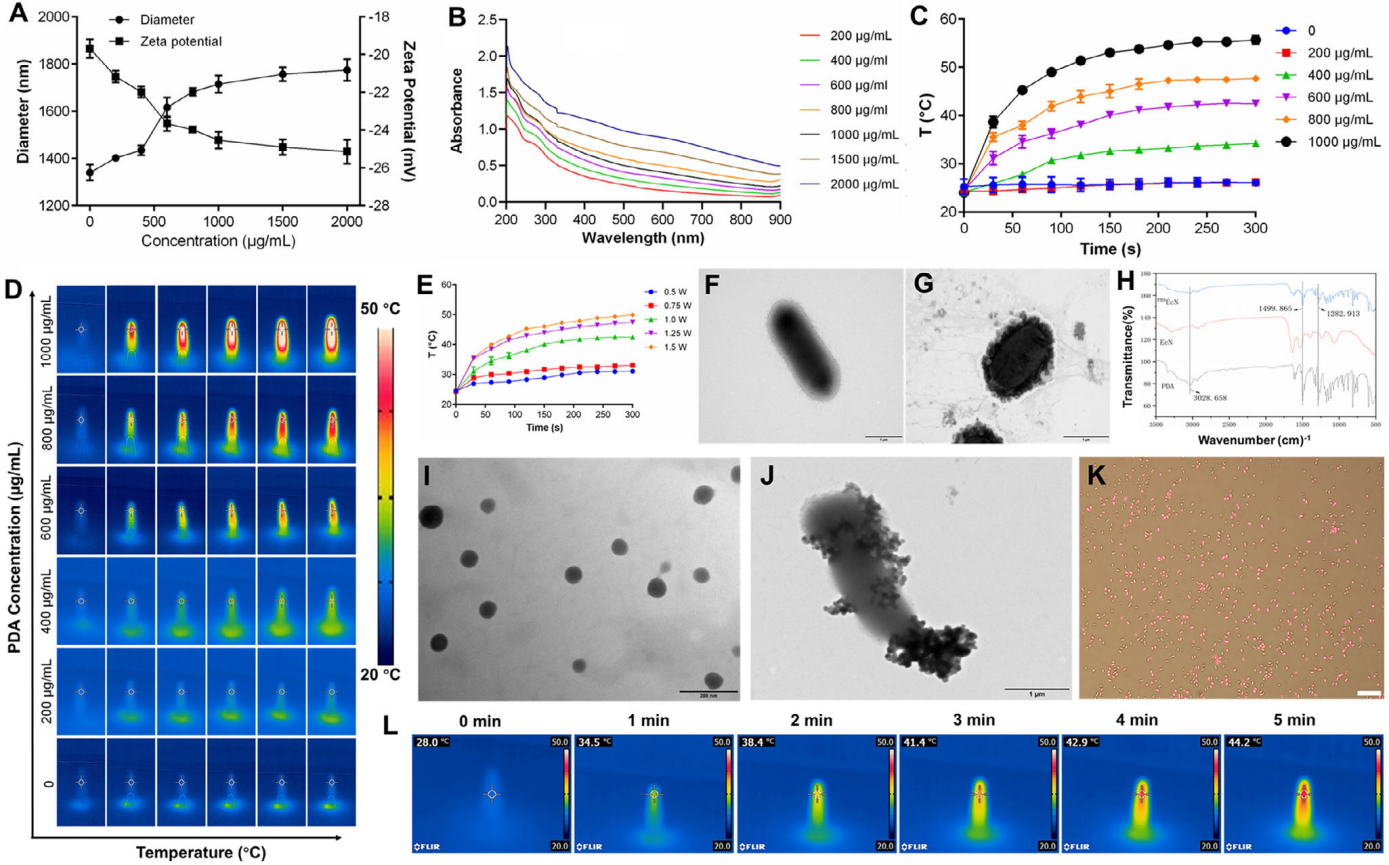
The particle size and zeta potential A), absorbance spectra B) of ^PDA^EcN prepared at 200–2000 µg mL^−1^ dopamine. Temperature change C) and photographs D) of deionized water or ^PDA^EcN prepared at various dopamine concentrations as a function of irradiation time with an 808 nm laser. Temperature change of ^PDA^EcN under various irradiation intensities as a function of irradiation time with an 808 nm laser E). TEM micrographs of EcN F) and ^PDA^EcN G). Scale bar = 1 µm. FTIR spectra of PDA, EcN, and ^PDA^EcN H). TEM images of COS NPs I) and ^PDA^EcN/COS J). The scale bar in figure I is 20 nm, and in figure J is 1 µm. Fluorescent image of ^PDA^EcN/COS@RHB. Scale bar = 10 µm K). Temperature photographs of ^PDA^EcN/COS as a function of irradiation time with an 808 nm laser L).

^PDA^EcN was synthesized in a single step through dopamine oxidation and self‐polymerization. The native EcN exhibited an average size of 1,340.4 nm and a zeta potential of −19.68 mV. In comparison, with the increase of dopamine concentration from 200 to 2,000 µg mL^−1^, ^PDA^EcN demonstrated an enlarged average size ranging from 1,402.1 to 1,774.2 nm and a zeta potential ranging from −21.17 to −25.14 mV, attributed to the greater number of hydroxyl groups formed by the thicker polydopamine coating (**Figure** **1**A). Concurrently, the color of the ^PDA^EcN suspension transitioned from clear to black as dopamine concentration elevated (Figure S1, Supporting Information). In addition, ^PDA^EcN exhibited broad absorption across 200–900 nm, with absorption intensity progressively increasing alongside dopamine concentration (200–2,000 µg mL^−1^, Figure 1B). Thicker polydopamine layers were thus formed at higher dopamine concentrations. Collectively, these findings confirmed the successful coating of PDA. The photothermal conversion efficiency of ^PDA^EcN was subsequently evaluated. Previous studies have indicated that high‐temperature PTT poses unavoidable risks to adjacent healthy tissues due to difficulties in limiting heat diffusion. Mild‐temperature PTT, maintaining temperatures below 45 °C, has been proposed as a promising strategy to avoid damage to normal cells. As shown in Figure 1C–E, the photothermal response of ^PDA^EcN under NIR irradiation exhibited a positive correlation with both dopamine concentration and irradiation intensity, indicating a dual‐parameter‐dependent thermal behavior. Upon increasing dopamine concentration to 600 µg mL^−1^, illumination at 1 W cm^−^^2^ led to an equilibrium temperature of 42.5 °C, appropriate for mild‐temperature PTT (Figure 1C,D). Specifically, solutions containing 600 µg mL^−1^ of dopamine were exposed to various laser powers of 0.5, 0.75, 1.0, 1.25, and 1.5 W cm^−^^2^ (Figure 1E). ^PDA^EcN exhibited a desirable temperature rise under a laser power of 1.0 W cm^−^^2^. Consequently, ^PDA^EcN prepared with 600 µg mL^−1^ dopamine and exposed to 1.0 W cm^−^^2^ laser power was selected as the optimal formulation for subsequent investigations. Transmission electron microscopy (TEM) revealed that native EcN exhibited a typical rod‐like morphology (Figure 1F), whereas the PDA coating was clearly visible on the EcN surface after 2 h of self‐polymerization (Figure 1G). Fourier transform infrared (FTIR) spectroscopy analysis of ^PDA^EcN showed multiple characteristic peaks between 1,000 and 1,700 cm^−1^ (Figure 1H), which corresponded closely to those observed in the PDA reference spectrum. In particular, peaks at 1,499 cm^−1^ (N–H shearing vibrations) and 1,282 cm^−1^ (C–OH stretching vibrations) were more pronounced. Additionally, a peak at 3,028 cm^−1^ was attributed to aromatic hydrogen stretching vibrations.

Subsequently, positively charged chitosan oligosaccharide (COS) and negatively charged polyacrylic acid (PAA) were employed to prepare COS NPs, as COS and PAA are capable of self‐assembling into nanoparticles in aqueous solutions via electrostatic interactions. As illustrated in Figure 1I, the morphology of the synthesized COS NPs exhibited a spherical structure with an average diameter of ≈150 nm determined by DLS analysis. The zeta potential analysis of the COS NPs revealed a value of about +25 mV, which can be ascribed to the high density of secondary and primary amine groups existing along the chitosan chains. ^PDA^EcN/COS was obtained by the deposition of COS NPs via electrostatic interactions with the negatively charged EcN surface. As illustrated in Figure 1J, COS NPs were successfully deposited onto the ^PDA^EcN surface, while the integrity of the ^PDA^EcN structure was preserved. Fluorescent COS@RHB was subsequently employed to further verify the binding affinity to ^PDA^EcN. Following incubation with COS@RHB, ^PDA^EcN exhibited strong red fluorescence, indicating successful adsorption onto its surface (Figure 1K). However, COS@RHB could hardly bind to the surface of EcN and exhibited almost invisible red fluorescence (Figure S2, Supporting Information).

The efficiency of photothermal conversion by ^PDA^EcN/COS was further evaluated. As illustrated in Figure 1L, the temperature of ^PDA^EcN/COS increased to 44.2 °C following 5 min of irradiation. It was postulated that the as‐prepared ^PDA^EcN/COS could readily induce heating to ≈45 °C within 5 min in vivo. Subsequently, the effects of 808 nm laser irradiation for 5 min on the viability of ^PDA^EcN/COS, ^PDA^EcN, and EcN were assessed. Minimal effects on the viability of ^PDA^EcN/COS were observed, as evidenced by the absence of significant red fluorescence (Figure S3, Supporting Information). Additionally, the potential impact of the PDA coating and COS NPs on the replication ability of EcN was investigated. The bacterial activity of ^PDA^EcN/COS was analyzed by inoculating it into LB culture medium and measuring bacterial counts at designated time points. In the case of ^PDA^EcN/COS, the exponential growth phase was delayed by more than 4 h compared to EcN, which proliferated rapidly. Nevertheless, both ^PDA^EcN/COS and EcN ultimately exhibited comparable proliferation rates (Figure S4, Supporting Information). ^PDA^EcN showed a similar proliferation curve to that of ^PDA^EcN/COS. These findings demonstrated that the PDA nanocoating binding could delay the growth phase of ^PDA^EcN/COS without impairing their bioactivity, thereby enhancing biosafety in vivo. Finally, the risk of PDA coating peeling under simulated physiological conditions was also investigated. As illustrated in Figure S5 (Supporting Information), the absorption intensity remained almost unchanged, which suggested a low risk of PDA coating peeling during the in vivo circulation.

### 2.2 Binding, Camouflaging, and Penetration Ability of ^PDA^EcN/COS

**Figure 2:**
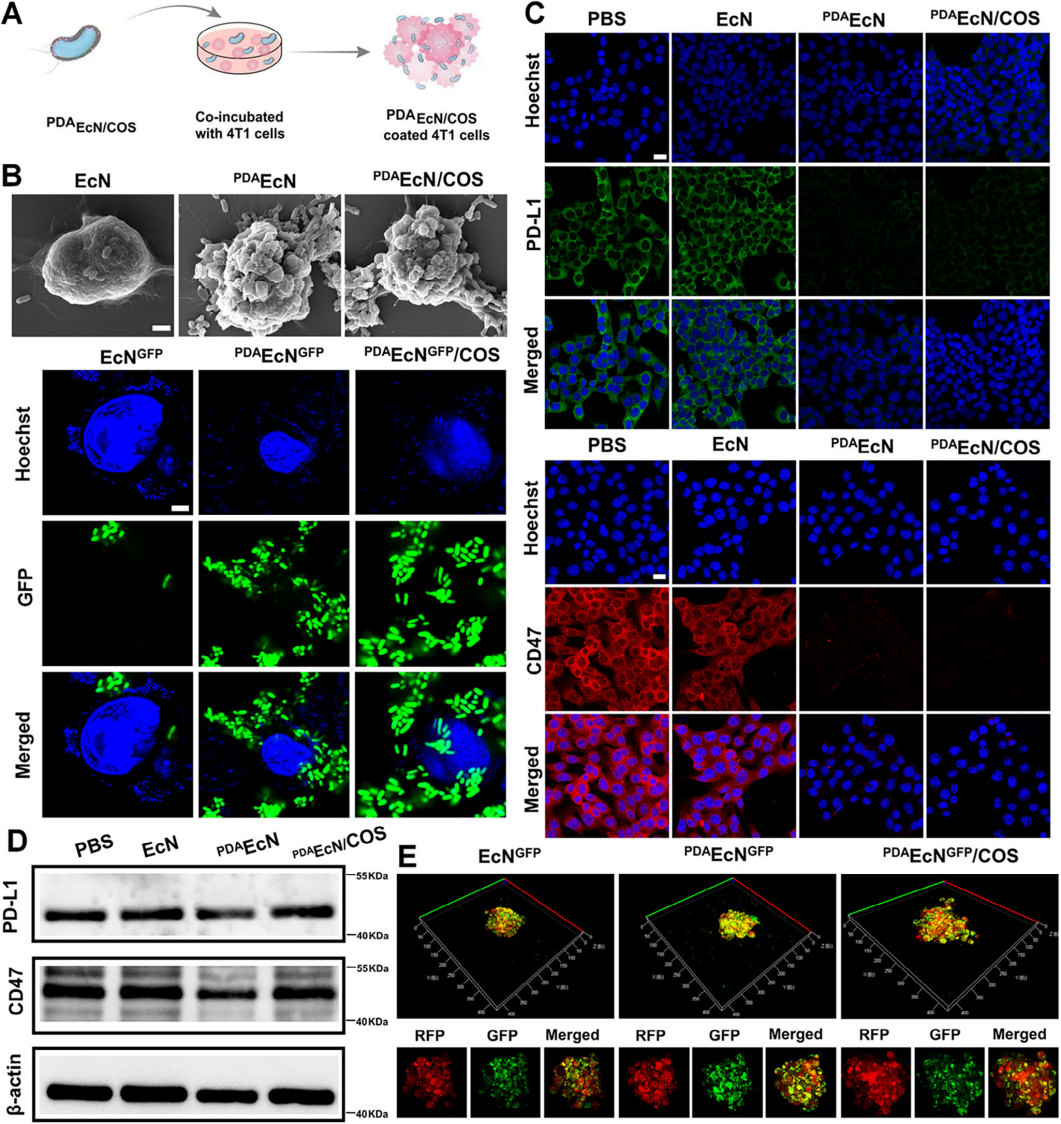
Schematic illustration of ^PDA^EcN/COS coverage of 4T1 cells A). SEM and CLSM images of 4T1 cells after co‐incubation with different formulations. Scale bar = 2 µm B). The IF assay C) and western blot analysis D) of PD‐L1 and CD47 expression in 4T1 cells after co‐incubation of PBS, EcN, ^PDA^EcN, and ^PDA^EcN/COS, respectively. Scale bar = 20 µm. Penetration of EcN^GFP^, ^PDA^EcN^GFP^, and ^PDA^EcN^GFP^/COS in MCSs after a 3 h incubation. The bottom images represented the fluorescence intensity of bacteria in the tumor core E).

In this study, it was hypothesized that ^PDA^EcN/COS could specifically target tumor cells, adhere to their surface, and effectively shield immune checkpoints (**Figure** **2**A). Therefore, the binding affinity of ^PDA^EcN/COS to 4T1 cells was initially evaluated and compared with that of EcN and ^PDA^EcN. SEM images revealed that a greater number of bacteria were observed to adhere to the tumor surface following treatment with PDA‐coated EcN (^PDA^EcN/COS and ^PDA^EcN) compared with non‐PDA‐coated EcN, indicating enhanced adhesive capability following PDA coating (Figure 2B). A similar result was obtained using fluorescent EcN observed by CLSM. A large number of fluorescent PAD‐coated bacteria surrounded the 4T1cells.

4T1 cells were found to overexpress PD‐L1 and CD47 on their membranes, enabling binding to PD‐1 on T lymphocytes and SIRPα on macrophages, thereby facilitating immune evasion. Blockade of the PD‐L1/PD‐1 and CD47/SIRPα pathways has previously been shown to activate antitumor immune responses. The aforementioned results suggested that PDA‐coated EcN could effectively adhere to the surface of 4T1 cells. It was hypothesized that bacterial coverage on the tumor cell surface could efficiently shield the exposure of PD‐L1 and CD47. Additionally, EcN located on the tumor surface might disguise tumor cells as bacteria, thus enhancing immune recognition and improving therapeutic efficacy. Consequently, the expression levels of PD‐L1 and CD47 on the surface of 4T1 cells following incubation with PBS, EcN, ^PDA^EcN, and ^PDA^EcN/COS were further assessed by IF. As illustrated in Figure 2C, the intensities of green and red fluorescence decreased after incubation with ^PDA^EcN and ^PDA^EcN/COS, indicating effective masking of PD‐L1 and CD47. Furthermore, PD‐L1 and CD47 expression levels under different treatments were also examined via western blotting (WB) assay, which demonstrated that there is no significant change in their overall expression levels (Figure 2D). These findings suggested that PDA‐coated EcN might effectively shield PD‐L1 and CD47 without significantly altering their intrinsic expression levels.

Subsequently, multicellular spheroids (MCSs) derived from 4T1^RFP^ cells were selected as an in vitro tumor model to assess the penetration ability of ^PDA^EcN/COS. In contrast to monolayer adherent cells, MCSs have emerged as a versatile 3D model for studying tumor biology and screening therapeutic agents. 4T1^RFP^ MCSs were incubated with ^PDA^EcN^GFP^/COS, EcN^GFP^, and ^PDA^EcN^GFP^ for 3 h, succeeded by washing and observation using CLSM (Figure 2E). For all treatments, green fluorescence signals from EcN were clearly detected within the MCSs, including the central regions, thereby demonstrating enhanced penetration of EcN. Furthermore, the coating with PDA and the loading of COS NPs did not impair the tumor‐penetrating ability of EcN.

### 2.3 Mild PTT In Vitro

#### 2.3.1 Cell Viability Assay

**Figure 3:**
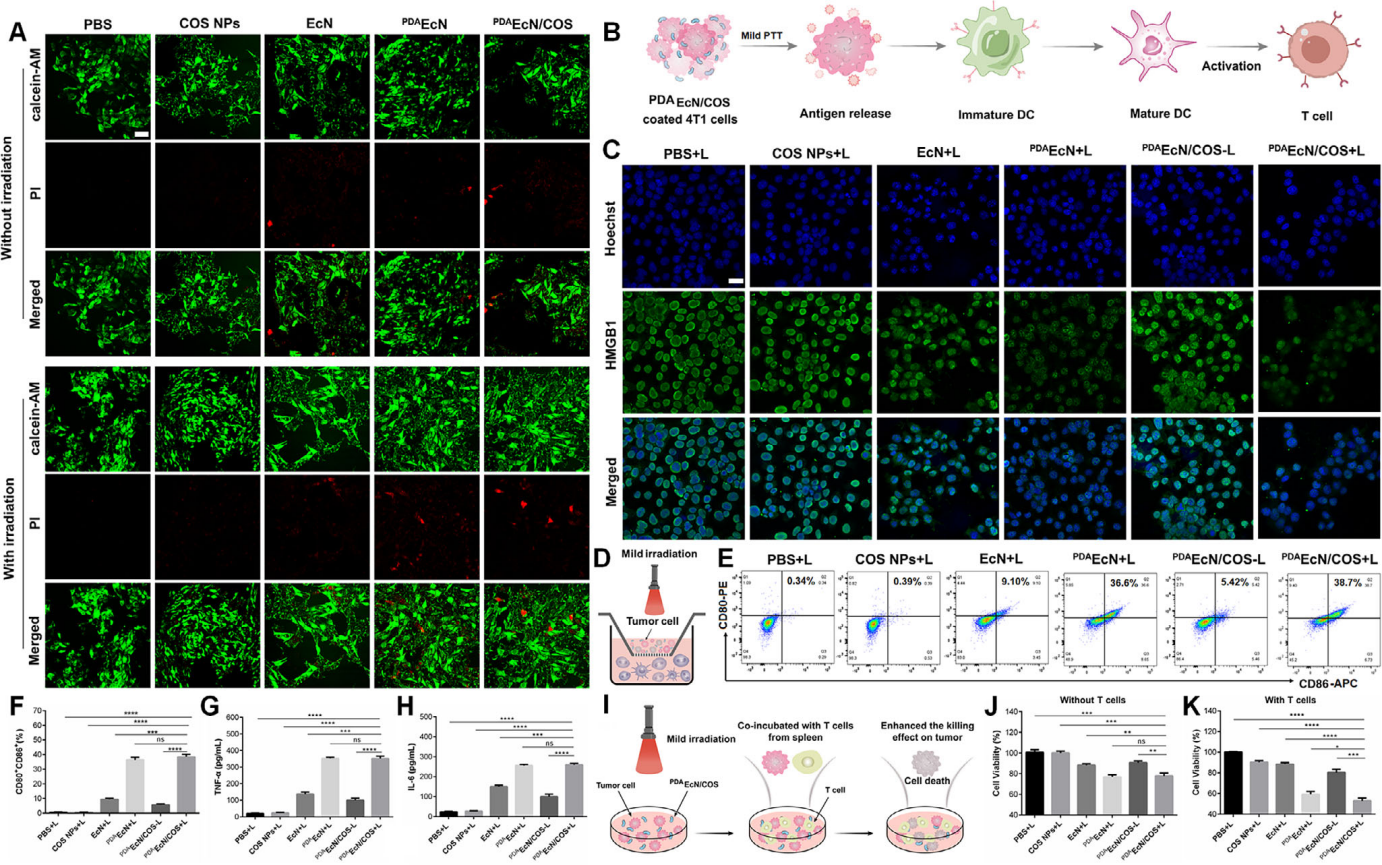
Live/dead assay of 4T1 cells after co‐incubation with PBS, COS NPs, EcN, ^PDA^EcN, and ^PDA^EcN/COS with and without irradiation. Scale bar = 50 µm A). Diagram depicting ICD triggered by mild PTT, resulting in elevated T lymphocyte populations within tumors, thereby stimulating anti‐cancer immune responses B). CLSM examination of HMGB1 expression in 4T1 cells exposed to various treatments. Scale bar = 20 µm C). Diagram illustrating the Transwell apparatus for mild PTT‐triggered ICD initiating photothermal‐mediated DC maturation D). Flow cytometric evaluation of CD80^+^CD86^+^ DC percentages across treatment groups E). CD80^+^CD86^+^ manifestation amongst various groups (n = 3), F). Quantitative determination of TNF‐α G) and IL‐6 H) cytokine concentrations in Transwell supernatants across different treatments. Diagram showing 4T1 cell survival following spleen cell exposure I). MTT‐based quantification of 4T1 cell survival rates without splenic T cells J) and in the presence of splenic T cells K). The mean values and S.D. were represented. ^*^p < 0.05, ^**^p < 0.01, ^***^p < 0.001, ^****^p < 0.0001, and ns represents no significant difference.

PTT has been recognized for its ability to facilitate efficient tumor eradication, characterized by noninvasiveness, controllability, and the induction of local hyperthermia. Furthermore, to minimize collateral thermal damage to adjacent healthy tissues and vital organs, the application of mild‐temperature PTT (below 45 °C) holds considerable clinical significance. As illustrated in **Figure** **3**A, in the absence of irradiation, ^PDA^EcN/COS, ^PDA^EcN, and EcN exhibited limited antitumor activity; nevertheless, their efficacy remained superior to that observed in the PBS group. Upon irradiation, the antitumor activities of both ^PDA^EcN/COS and ^PDA^EcN were moderately enhanced, as evidenced by a slight increase in red fluorescence intensity compared to their non‐irradiated counterparts, although the majority of tumor cells remained viable. Therefore, mild PTT at relatively low temperatures was employed as an adjunctive approach in tumor treatment rather than as a direct cytotoxic modality. Additionally, COS NPs demonstrated no observable antitumor activity either with or without irradiation.

#### 2.3.2 Photothermal Induces Immune Activation In Vitro

The induction of mild PTT was anticipated to trigger ICD, which was expected to alleviate tumor‐associated immunosuppression by promoting the exposure of DAMPs, such as calreticulin and HMGB1, thereby enhancing DC maturation, improving antigen presentation, and activating T cells, ultimately stimulating an antitumor immune response and leading to tumor cell elimination (Figure 3B). Given that the surface of 4T1 cells was masked by PDA‐coated EcN, only the intracellular levels of HMGB1 were assessed after various treatments. As illustrated in Figure 3C, intracellular HMGB1 fluorescence intensity was reduced in both the ^PDA^EcN/COS and ^PDA^EcN groups, thereby confirming that the mild photothermal effect could induce ICD in cancer cells and release DAMPs.

Subsequently, DC maturation, characterized by the upregulation of costimulatory molecules CD80 and CD86, was examined. As illustrated by the Transwell experimental design shown in Figure 3D, DCs situated in the lower chamber underwent collection to analyze maturation markers through FCM. Compared with the PBS control group, EcN treatment resulted in a slightly higher proportion of matured DCs (CD86⁺CD80⁺, 9.36 ± 0.74%). Moreover, with the assistance of PDA coating, the groups treated with ^PDA^EcN/COS+L and ^PDA^EcN+L exhibited a markedly elevated DC maturation rate exceeding 35%, likely attributable to the mild photothermal effect of PDA (Figure 3E,F). It was also observed that the maturation rate in the ^PDA^EcN/COS‐L group remained ≈5%, notably lower than that of the EcN group. It was hypothesized that the PDA nanocoating could reduce the cytotoxicity of EcN toward 4T1 cells, thereby diminishing the release of HMGB1. In addition, ICD induced by ^PDA^EcN/COS and ^PDA^EcN markedly enhanced the secretion of TNF‐α and IL‐6, two essential immune cytokines associated with DC maturation (Figure 3G,H). Collectively, these findings demonstrated that the mild photothermal effect of the PDA nanocoating effectively induced ICD, thereby promoting DC maturation and activating the antitumor immune response.

To verify whether mild‐temperature pre‐irradiation could enhance the ability of T cells to eliminate tumor cells, the experimental design is depicted in Figure 3I. The viability of pre‐irradiated 4T1 cells, incubated with or without spleen cells, was assessed using the MTT assay. In the absence of spleen cells, the viability of 4T1 cells treated with ^PDA^EcN/COS+L was slightly reduced to 77.9% versus the blank irradiation group (Figure 3J), consistent with results from the live/dead assay (Figure 3A). Notably, following incubation with spleen cells, the viability of 4T1 cells treated with ^PDA^EcN/COS under irradiation was markedly decreased by 52.9%, while without irradiation, it was 80.23% (Figure 3K). This enhanced tumor cell‐killing effect mediated by T cells may be attributed to the mild photothermal effect of the PDA nanocoating, which could effectively induce tumor ICD, subsequently activate T cells, which might reprogram the tumor TME.

### 2.4 Macrophage Polarization

#### 2.4.1 Pro‐Inflammatory Immunomodulatory

**Figure 4:**
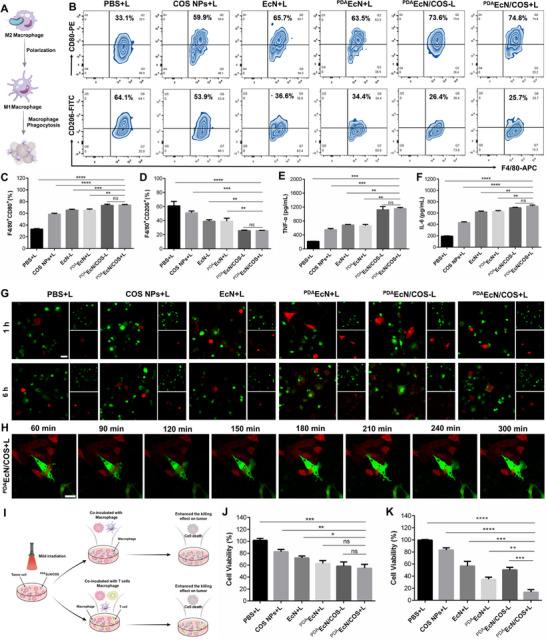
Schematic illustration of EcN and COS NPs polarization of M2 macrophages to M1 macrophages A). FCM analysis of the proportion of M1 macrophages (labelled with F4/80^+^CD80^+^) and M2 macrophages (labelled with F4/80^+^CD206^+^) B). Percentages of F4/80^+^CD80^+^ C) and F4/80^+^CD206^+^ D) cells. Cytokine content of TNF‐α E) and IL‐6 F) tested by ELISA. Representative CLSM images of phagocytosis assays treated with different formulations for 1 h and 6 h, respectively G). Time‐dependent phagocytosis treated with ^PDA^EcN/COS+L H). In (G) and (H), macrophages were labelled with GFP (green) and 4T1 cells were labelled with RFP (red). Scale bar = 20 µm. Schematic illustration of 4T1 cell viability incubated with macrophages or the mixture of splenic T cells and macrophages I). Quantitative analysis of 4T1 cell viability measured by MTT assay with macrophages J) and with macrophages and splenic T cells K). The mean values and S.D. were represented. ^*^p < 0.05, ^**^p < 0.01, ^***^p < 0.001, ^****^p < 0.0001, and ns represents no significant difference.

Macrophages, a critical class of immune cells, are known to serve essential functions during the resolution phase of inflammation and tissue regeneration. In mammals, macrophages can undergo polarization into pro‐inflammatory M1 or anti‐inflammatory M2 phenotypes depending on specific microenvironmental stimuli. It has been reported that COS NPs are capable of activating macrophages and triggering the release of pro‐inflammatory cytokines, thereby inducing an appropriate immune response against tumors. Furthermore, owing to the abundance of pathogen‐associated molecular patterns within bacteria, immune cell activation can be effectively achieved even within tumor‐immunosuppressive microenvironments, enhancing specific immune recognition and subsequent elimination of tumor cells. Thus, both COS NPs and EcN presented in ^PDA^EcN/COS could induce M2 macrophage polarization (**Figure** **4**A). To evaluate the impact of ^PDA^EcN/COS on macrophage polarization, IL‐4 was initially added to mouse peritoneal‐derived macrophages to induce M2 polarization, succeeded by incubation with diverse formulations and subsequent analysis by FCM. The expression of F4/80⁺ CD80⁺ (M1 marker) and F4/80⁺ CD206⁺ (M2 marker) was assessed independently on different batches of macrophages. In contrast to the control group, treatments with COS NPs+L, EcN+L, ^PDA^EcN+L, ^PDA^EcN/COS‐L, and ^PDA^EcN/COS+L markedly increased the proportion of M1 macrophages (F4/80⁺ CD80⁺) and decreased that of M2 macrophages (F4/80⁺ CD206⁺), indicating a successful repolarization from the M2 to M1 phenotype (Figure 4B). Notably, macrophages treated with ^PDA^EcN/COS+L exhibited a high M1 phenotype (74.2 ± 0.78%) and a low M2 phenotype (25.97 ± 0.25%), compared with COS NPs+L (58.5 ± 1.51% and 51.2 ± 2.52%, respectively) and EcN+L (65.57 ± 1.4% and 39.17 ± 2.24%, respectively) groups (Figure 4C,D). These observations suggested that the combination of COS NPs and EcN could synergistically facilitate the conversion of M2 macrophages into the M1 phenotype. Moreover, no notable distinction was detected between the ^PDA^EcN/COS‐L and ^PDA^EcN/COS+L groups regarding M1 and M2 phenotypes, suggesting that mild PTT exerted limited effects on macrophage polarization. To further investigate macrophage polarization, an ELISA kit was employed to measure the secretion levels of TNF‐α and IL‐6 by M1 macrophages. As depicted in Figure 4E,F, treatments with COS NPs, EcN+L, and ^PDA^EcN/COS+L markedly upregulated the secretion of both TNF‐α and IL‐6. Furthermore, the combination of COS NPs and EcN exhibited a pronounced co‐polarization effect, leading to 5.37‐ and 3.77‐fold increases in TNF‐α and IL‐6 levels, respectively, compared with the control group. These findings further confirmed the effective polarization from M2 to M1 induced by the systems. Given the pivotal role of TAMs within the TME, their repolarization is expected to markedly enhance the efficacy of immunotherapy and inhibit tumor growth and progression.

#### 2.4.2 Macrophage Phagocytosis Assay

Building upon the M1 phenotype polarization induced by ^PDA^EcN/COS+L, the phagocytosis of 4T1 cells by RAW264.7 macrophages following treatment was further examined, with 4T1 and RAW264.7 cells exhibiting red and green fluorescence, respectively. As illustrated in Figure 4G, a greater number of macrophages were observed to migrate toward tumor cells, surrounding and initiating phagocytosis after 1 h of co‐incubation with PDA‐coated *E. coli* compared to other groups. Upon extending the incubation period to 6 h, a marked increase in 4T1 cell phagocytosis by macrophages was evident in the ^PDA^EcN+L, ^PDA^EcN/COS‐L, and ^PDA^EcN/COS+L groups relative to other treatments, including EcN+L. These findings indicated that the PDA coating facilitated effective adhesion of EcN onto the tumor cell surface, thereby shielding the surface CD47 of tumor cells and enhancing the phagocytosis of the 4T1‐EcN complex by macrophages. To further elucidate this process, time‐course tracking of phagocytosis was conducted. As depicted in Figure 4H, RAW264.7 cells were observed to progressively contact and ultimately phagocytose 4T1 cells following treatment with ^PDA^EcN/COS+L.

#### 2.4.3 In Vitro Antitumor Activity

The antitumor efficacy of the combination therapy was further evaluated, with the experimental design illustrated in Figure 4I. The viability of pre‐irradiated 4T1 cells, following incubation with either macrophages alone or a mixture of macrophages and T cells, was assessed using the MTT assay. As depicted in Figure 4J, the cell viability for the COS NPs+L, EcN+L, ^PDA^EcN+L, ^PDA^EcN/COS‐L, and ^PDA^EcN/COS+L groups in the presence of macrophages was recorded as 83.2 ± 3.63%, 72.0 ± 3.39%, 62.8 ± 4.83%, 58.4 ± 6.82%, and 54.9 ± 6.42%, respectively. It is noteworthy that no significant difference in antitumor activity was observed between the ^PDA^EcN/COS‐L and ^PDA^EcN/COS+L groups, indicating that mild PTT alone did not induce macrophage polarization or promote phagocytosis. Upon the further addition of T cells, the cell viability of ^PDA^EcN/COS+L‐treated 4T1 cells decreased markedly to 13.57 ± 4.11%, suggesting a robust antitumor effect (Figure 4K). Moreover, the antitumor activity of ^PDA^EcN/COS in the presence of irradiation was found to be markedly greater than that in its absence, suggesting a synergistic enhancement arising from T cell activation by mild PTT combined with macrophage polarization induced by COS NPs and EcN.

### 2.5 In Vivo Systemic Inflammatory Response of ^PDA^EcN/COS

**Figure 5:**
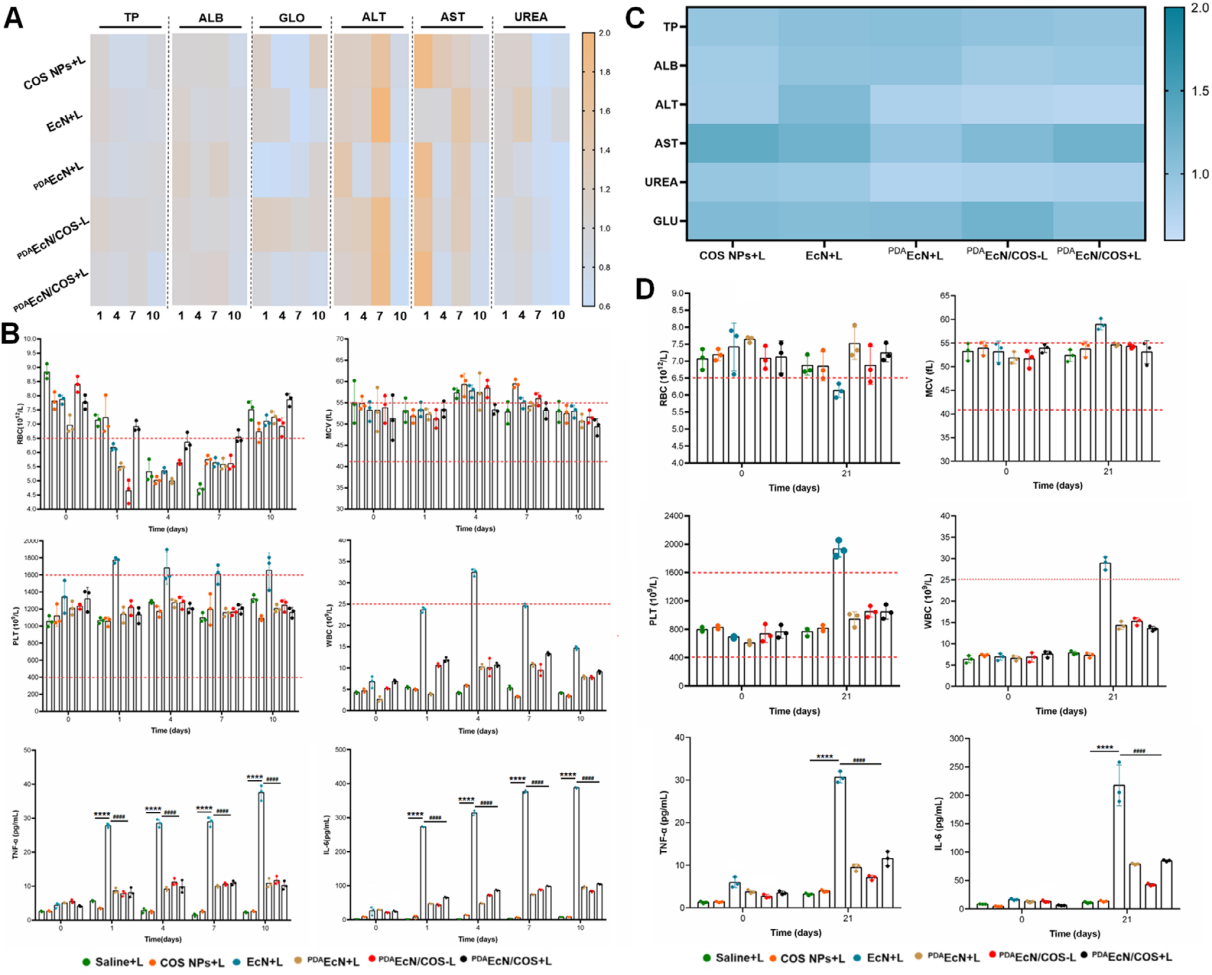
Systemic inflammatory response and long‐time safety test of ^PDA^EcN/COS in vivo. Evaluation of liver and kidney functions via biochemistry test in different groups at day 1, 4, 7, and 10 days after injection A). Routine blood analysis, including RBCs, MCV, PLTs, WBCs TNF‐α and IL‐6 in healthy mice on different days for postinjection B). Blood biochemistry test in different groups before and after three administrations C). Blood routine analysis in different groups before and after three administrations D). There were three mice in each group. The mean values and S.D. were represented. ^****^p <0.0001, markedly different from saline+L. *^####^P*<0.0001, markedly different from ^PDA^EcN/COS+L.

Given that the immune activation efficacy of ^PDA^EcN/COS had been confirmed in vitro, an evaluation was executed to determine whether systemic inflammatory responses would be induced upon intravenous administration. This assessment aimed to examine the biosafety and biocompatibility of ^PDA^EcN/COS and to inform its potential for clinical translation. Murine blood samples were collected at 1, 4, 7, and 10 days post‐injection and analyzed using biochemical assays and a blood routine analyzer. Compared with the EcN‐treated group, most biochemical parameters in the ^PDA^EcN/COS‐treated mice remained within normal ranges throughout the 10 days, indicating that ^PDA^EcN/COS did not cause detectable renal or hepatic toxicity in vivo (**Figure** **5**A). It was observed that red blood cell (RBC) counts were reduced and mean corpuscular volume (MCV) levels were elevated on days 4 and 7 compared with other time points across all treated groups. This phenomenon was speculated to be related to the frequency of blood sampling. By day 10, RBC counts and MCV values had returned to baseline levels, suggesting that any transient anemia had been resolved. Furthermore, it was noted that mice administered EcN, at a bacterial dosage equivalent to that used for ^PDA^EcN/COS and ^PDA^EcN groups, exhibited slight elevations in platelet (PLT) and white blood cell (WBC) counts during systemic circulation at 1, 4, 7, and 10 days, and particularly at 4 and 7 days post‐injection. Additionally, the measurement of IL‐6 and TNF‐α levels also confirmed the induction of systemic inflammation in the EcN‐treated group (Figure 5B).

Subsequently, the long‐term safety of ^PDA^EcN/COS was evaluated following repeated administrations. Consistent with the results of the acute toxicity study, no significant abnormalities in blood samples (Figure 5C,D) or organ histology (Figure S6, Supporting Information) were observed after three injections administered over 21 days, except in the EcN‐treated group. These findings indicated that the PDA‐coated EcN formulations exhibited excellent in vivo biocompatibility, suggesting suitability for clinical applications. Furthermore, the results revealed that constructing bacteria‐based delivery systems coated with PDA could prevent the induction of systemic inflammation compared with uncoated bacteria. It was speculated that the PDA nanocoating might shield immunogenic bacterial surface antigens. To validate this hypothesis, the expression of LPS on the surfaces of EcN, ^PDA^EcN, and ^PDA^EcN/COS was further examined. As illustrated in Figure S7 (Supporting Information), uncoated EcN displayed strong red fluorescence, indicating a high surface expression of LPS. In contrast, PDA‐coated EcN exhibited much weaker red fluorescence, suggesting a reduction in LPS expression. Therefore, coating bacteria with PDA nanolayers could not only enhance their adhesion to tumor cells and enable mild PTT but also suppress LPS expression, thereby reducing their immunogenicity in vivo.

### 2.6 In Vivo Biodistribution and Photothermal Effect

**Figure 6:**
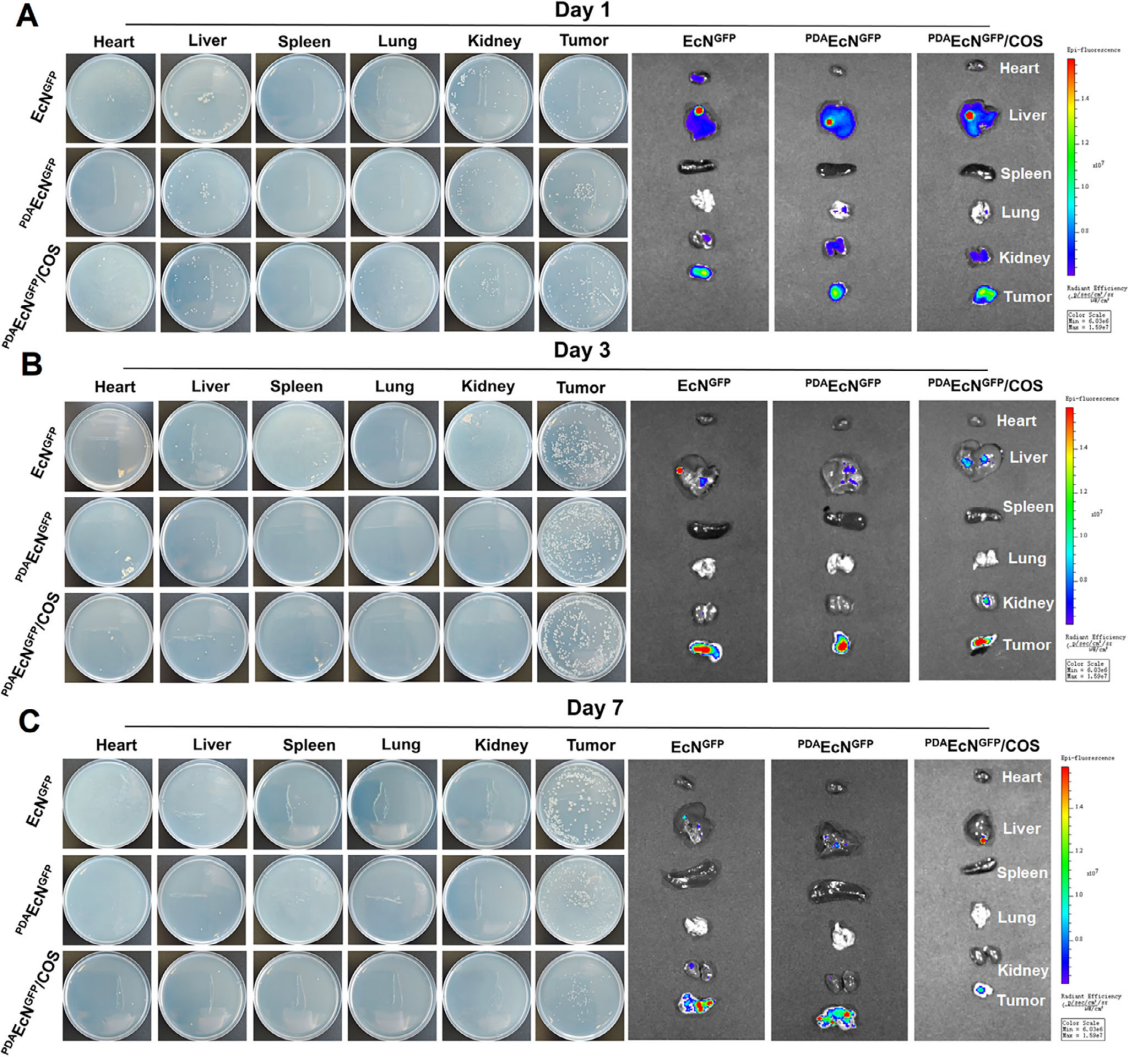
In vivo biodistribution of ^PDA^EcN^GFP^/COS. Selective growth and fluorescence intensity of EcN^GFP^, ^PDA^EcN^GFP,^ and ^PDA^EcN^GFP^/COS in tumors and in heart, liver, spleen, lung, and kidney on day 1 A), day 3 B) and day 7 C).

Moreover, the in vivo biodistribution of ^PDA^EcN^GFP^/COS was investigated in mice bearing 4T1 tumors. The heart, liver, spleen, lung, kidney, and tumor tissues were imaged at various time points post‐injection, subsequently homogenized, and plated onto LB agar plates for bacterial enumeration. On the first day following injection, bacterial presence was detected in most organs, including the tumor sites (**Figure** **6**A; Figure S8A, Supporting Information). As time progressed, both the fluorescence intensity and bacterial counts in normal tissues decreased steadily, whereas bacterial accumulation within the tumor tissue increased and remained relatively high up to three days post‐injection (Figure 6B; Figure S8B, Supporting Information). Furthermore, no significant difference was observed between EcN^GFP^ and ^PDA^EcN^GFP^/COS, suggesting that PDA coating did not impair the targeting ability or proliferative capacity of EcN. The preferential accumulation of these bacteria within tumor tissues was likely attributable to their affinity for the hypoxic environment and immune suppression characteristic of the TME, which protected them from immune clearance. These findings indicated that functional ^PDA^EcN^GFP^/COS exhibited both specific localization and proliferative capacity within tumor sites. However, by day 7 post‐injection, both fluorescence signals and bacterial numbers (Figure 6C; Figure S8C, Supporting Information) in the ^PDA^EcN/COS‐treated group were lower than those in the EcN^GFP^‐treated group and ^PDA^EcN^GFP^‐treated group. Additionally, the tumor size in the ^PDA^EcN^GFP^/COS group was smaller than that observed in the other two groups. This result might be attributed to the immune activation effect elicited by ^PDA^EcN^GFP^/COS, which contributed to tumor suppression.

The intratumor photothermal conversion of ^PDA^EcN/COS was also investigated. Infrared thermal images and the corresponding temperature variations of the tumor regions in mice bearing 4T1 tumor models were recorded. A rapid temperature increase at the tumor site was observed following NIR irradiation on day 3 post‐injection, with ^PDA^EcN/COS enabling the maintenance of a relatively low temperature of ≈45 °C (Figure S9, Supporting Information).

### 2.7 In Vivo Antitumor Efficacy of ^PDA^EcN/COS

**Figure 7:**
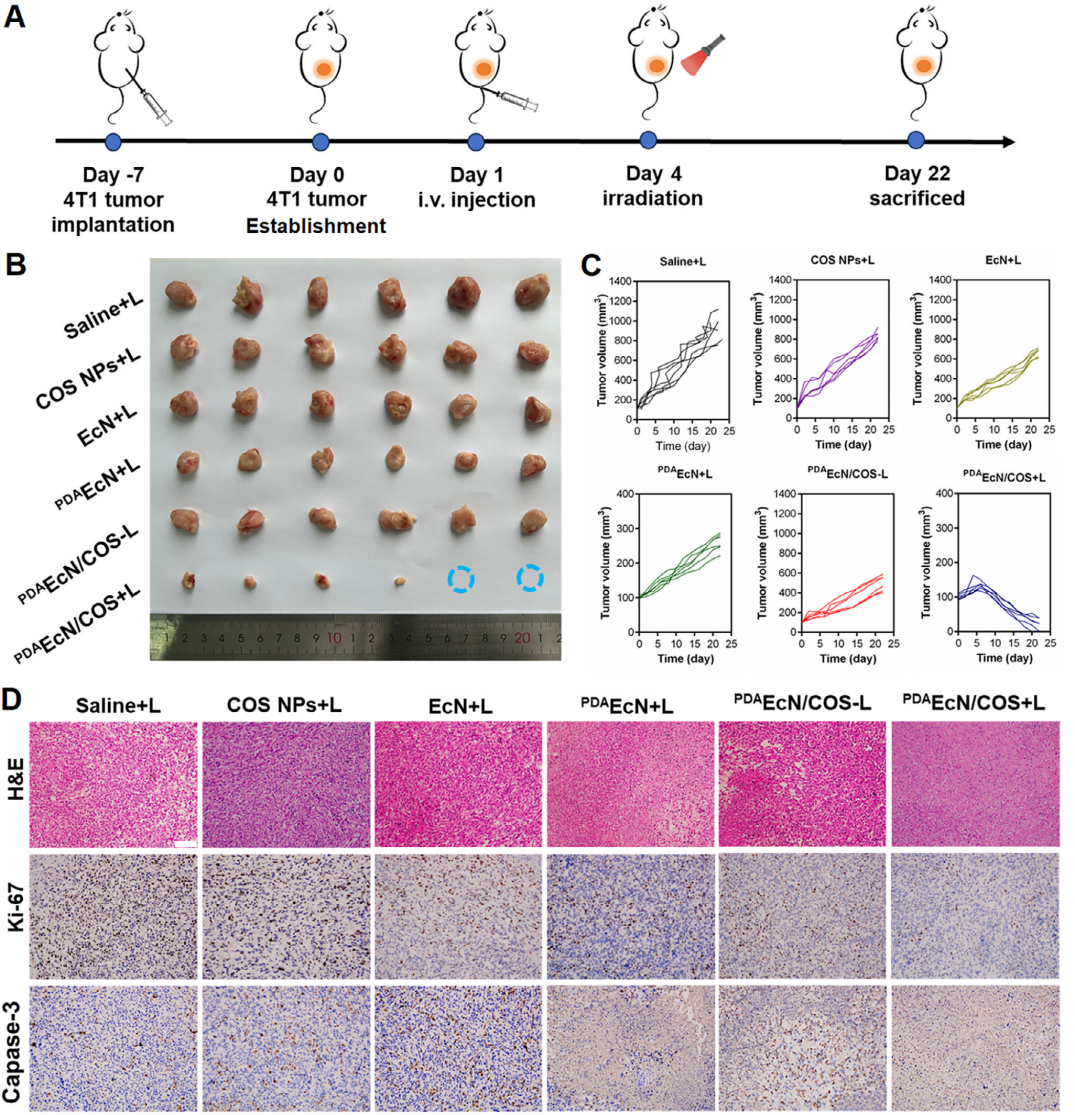
Illustration of the animal experimental design A). Tumor photos B), tumor growth curves C) of 4T1 tumor‐bearing BALB/c mice injected with different formulations (n = 6). H&E, Ki‐67, and Capase‐3 staining images of the tumor tissues after different treatments (n = 3). Scar bar = 50 µm.

The therapeutic efficacy of ^PDA^EcN/COS was subsequently evaluated in a subcutaneous 4T1 murine model (**Figure** **7**A). Following transplantation of 4T1 tumor cells for seven days, the mice were randomly allocated into six groups and injected with saline+L, COS NPs+L, EcN+L, ^PDA^EcN+L, ^PDA^EcN/COS‐L, or ^PDA^EcN/COS+L, respectively. Each formulation was administered intravenously once on day 1, with NIR irradiation applied on day 4 post‐injection. Body weights were recorded every two days over 22 days. According to Figure 7B,C, the ^PDA^EcN/COS+L group exhibited the slowest tumor progression and the smallest tumor volumes, achieving a 97.7% inhibition rate compared with the saline group (Figure S10A, Supporting Information). In contrast, mice treated with EcN+L displayed only limited therapeutic efficacy in inhibiting tumor growth, indicating that bacterial administration without the adhesive nanocoating, although capable of polarizing macrophages toward the M1 phenotype, was insufficient to inhibit solid tumor growth. Furthermore, both ^PDA^EcN+L and ^PDA^EcN/COS‐L treatments demonstrated weaker antitumor efficacy relative to ^PDA^EcN/COS+L, thereby underscoring the immune‐activating capabilities of COS NPs and the contribution of mild PTT. These findings emphasize the significance of surface adhesion properties and the synergistic effects of mild photothermal immunomodulation, macrophages polarization, and bacterial therapy in enhancing tumor inhibition. No significant body weight loss was observed, suggesting that ^PDA^EcN/COS+L was well tolerated (Figure S10B, Supporting Information). Subsequently, histological analyses using haematoxylin and eosin (H&E) staining, together with immunohistochemical staining for Ki‐67 and caspase‐3, confirmed that ^PDA^EcN/COS+L treatment led to significant tumor growth suppression (Figure 7D). Specifically, H&E staining revealed that ^PDA^EcN/COS+L induced more extensive tumor apoptosis and necrosis compared with other groups. Additionally, consistent with tumor growth results, tumor slices from mice treated with ^PDA^EcN/COS+L exhibited the lowest Ki‐67 expression and the highest caspase‐3 levels, thereby indicating the strong anti‐proliferative potential of ^PDA^EcN/COS under irradiation.

### 2.8 ^PDA^EcN/COS turns “Cold” Tumors “Hot”

**Figure 8:**
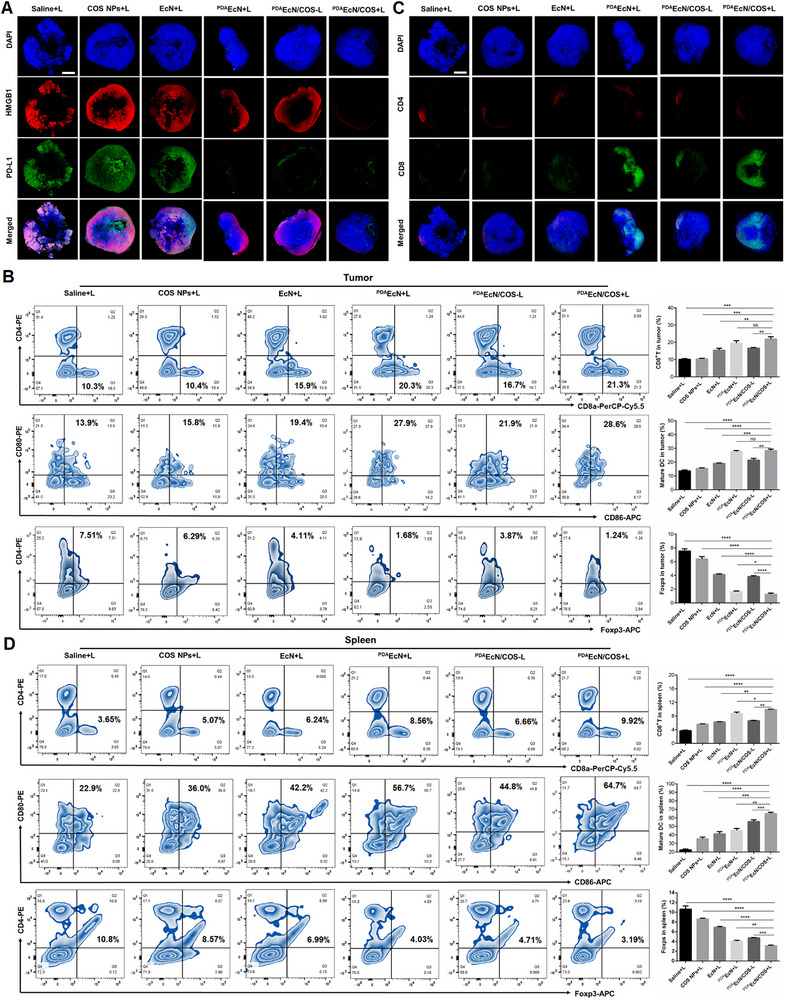
IF staining of the tumor slices after different treatments (Nuclei, blue fluorescence; HMGB1, red fluorescence; PD‐L1, green fluorescence). Scale bar = 1000 µm A). The FCM quantitative analysis for CD8^+^ T cells infiltration (CD45^+^CD4^−^CD8^+^), DCs maturation (CD11c^+^CD80^+^CD86^+^), and Treg cells (CD45^+^CD4^+^Foxp3^+^) in tumors B). IF staining of the tumor slices after different treatments (Nuclei, blue fluorescence; CD4^+^, red fluorescence; CD8^+^, green fluorescence). Scale bar = 1000 µm C). FCM quantitative analysis for CD8^+^ T cells infiltration (CD45^+^CD4^−^CD8^+^), DCs maturation (CD11c^+^CD80^+^CD86^+^), and Treg cells (CD45^+^CD4^+^Foxp3^+^) in spleens D). The mean values and S.D. were represented (n = 3). ^*^p < 0.05, ^**^p < 0.01, ^***^p < 0.001, ^****^p < 0.0001, and ns represents no significant difference.

The capacity of ^PDA^EcN/COS under irradiation to remodel the tumor immunosuppressive microenvironment was subsequently assessed. Tumor tissues and spleens were harvested following a single intravenous injection of saline+L, COS NPs+L, EcN+L, ^PDA^EcN+L, ^PDA^EcN/COS‐L, or ^PDA^EcN/COS+L, respectively. Initially, the exposure of HMGB1 was investigated through IF staining. As depicted in **Figure** **8**A, HMGB1 (red fluorescence) was predominantly co‐localized with nuclei (blue) in tumor tissues obtained from the saline‐treated group, whereas a marked reduction in nuclear HMGB1 was observed in the ^PDA^EcN/COS+L‐treated group. This suggested that ^PDA^EcN/COS+L promoted greater translocation and release of HMGB1, thereby activating the ICD effect in vivo. Additionally, the shielding of the immune checkpoint capability was also observed. The tumor slices from groups treated with ^PDA^EcN‐containing formulations exhibited substantially reduced green fluorescence intensity, indicating lower PD‐L1 exposure. This finding implied that ^PDA^EcN could effectively adhere to the tumor surface and obscure PD‐L1.

Based on the aforementioned analysis, it was proposed that ^PDA^EcN/COS+L might potentiate the antitumor immune response through a synergistic mechanism involving the induction of ICD. To validate this hypothesis, tumors and spleen tissues were collected after treatment for immune cell population analysis via FCM. The results demonstrated that ^PDA^EcN/COS+L treatment markedly enhanced the infiltration of CD8^+^ T cells (gated on CD45^+^) within the tumor tissue (Figure 8B). Furthermore, IF staining of tumor slices corroborated this tendency (Figure 8C). Given that matured DCs are capable of presenting antigens to CTLs, which are pivotal in eliciting anti‐tumor specific immune responses, the alterations in DCs within tumor tissues were subsequently analyzed by FCM. The findings revealed that the proportion of matured DCs (CD80^+^ CD86^+^ cells gated on CD11c^+^) was markedly elevated in the ^PDA^EcN/COS+L group, whereas the population of regulatory T cells (Treg, Foxp3^+^ CD4^+^ cells gated on CD45^+^) was substantially reduced (Figure 8B). In addition, immune profiling of the spleen demonstrated that the proportion of matured DCs in the ^PDA^EcN/COS+L group reached 64.7%, ≈2.82‐fold higher than that observed in the saline group (22.9%, Figure 8D), suggesting that the DAMPs exposure induced by ^PDA^EcN/COS+L could effectively promote DC maturation within the spleen. Moreover, a notable increase in the populations of CD8^+^ T cells and a concurrent reduction in Treg cells were consistently observed following ^PDA^EcN/COS+L treatment.

**Figure 9:**
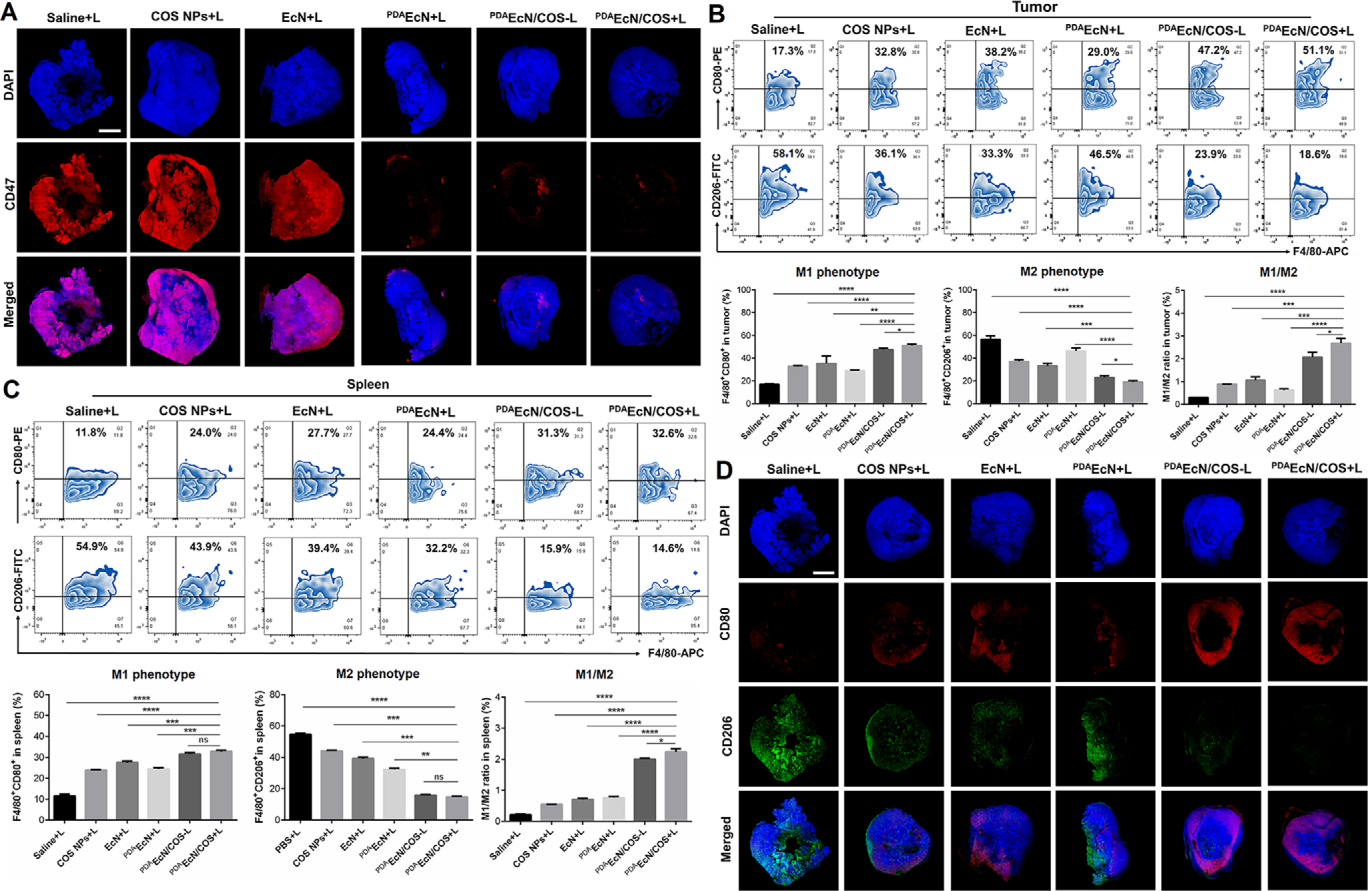
IF staining of the tumor slices after different treatments (Nuclei, blue fluorescence; CD47, red fluorescence). Scale bar = 1000 µm A). The FCM quantitative analysis of the proportion of M1 macrophages (F4/80^+^CD80^+^), M2 macrophages (F4/80^+^CD206^+^), percentages of F4/80^+^CD80^+^, F4/80^+^CD206^+,^ and M1/M2 in tumors B) and spleens C). IF staining of the tumor slices after different treatments (Nuclei, blue fluorescence; CD80, red fluorescence; CD206, green fluorescence). Scale bar = 1000 µm D). The mean values and S.D. were represented (n = 3). ^*^p < 0.05, ^**^p < 0.01, ^***^p < 0.001, ^****^p < 0.0001, and ns represents no significant difference.

Subsequently, the exposure of CD47 in tumor slices following various treatments was examined. As illustrated in **Figure** **9**A, markedly reduced red fluorescence intensity was observed in tumor slices treated with ^PDA^EcN+L, ^PDA^EcN/COS‐L, and ^PDA^EcN/COS+L, suggesting a decreased exposure of CD47. This observation was consistent with the findings related to PD‐L1 exposure, implying that PDA‐coated EcN could efficiently adhere to the tumor cell surface, thereby masking immune checkpoints and enhancing the recognition of tumor cells by T cells and macrophages. Finally, the macrophage phenotype within both tumor and spleen tissues was assessed. Notably, the administration of ^PDA^EcN/COS+L resulted in an increase in the expression of the M1 marker CD80 from 17.3% to 51.1% within tumors (Figure 9B) and from 11.8% to 32.6% within spleens (Figure 9C). Concurrently, ^PDA^EcN/COS+L induced a reduction in CD206 expression from 58.1% to 18.6% in tumors and from 54.9% to 14.6% in spleens. Furthermore, the M1/M2 ratio in both tumors and spleens was markedly elevated compared with that observed in the saline‐treated group. These findings demonstrated that ^PDA^EcN/COS+L was capable of diminishing the proportion of TAMs within the TME, thereby substantially enhancing the therapeutic efficacy of immunotherapy. IF staining of CD206 and CD80 further corroborated these observations (Figure 9D). Collectively, these results suggested that ^PDA^EcN/COS+L could markedly potentiate anti‐tumor immunotherapy through the synergistic mechanisms of ICD activation, macrophage polarization, and immune checkpoint shielding, turning “cold” tumors “hot”.

### 2.9 ^PDA^EcN/COS Remodeling the TME Landscape to “Hot” Tumors

**Figure 10:**
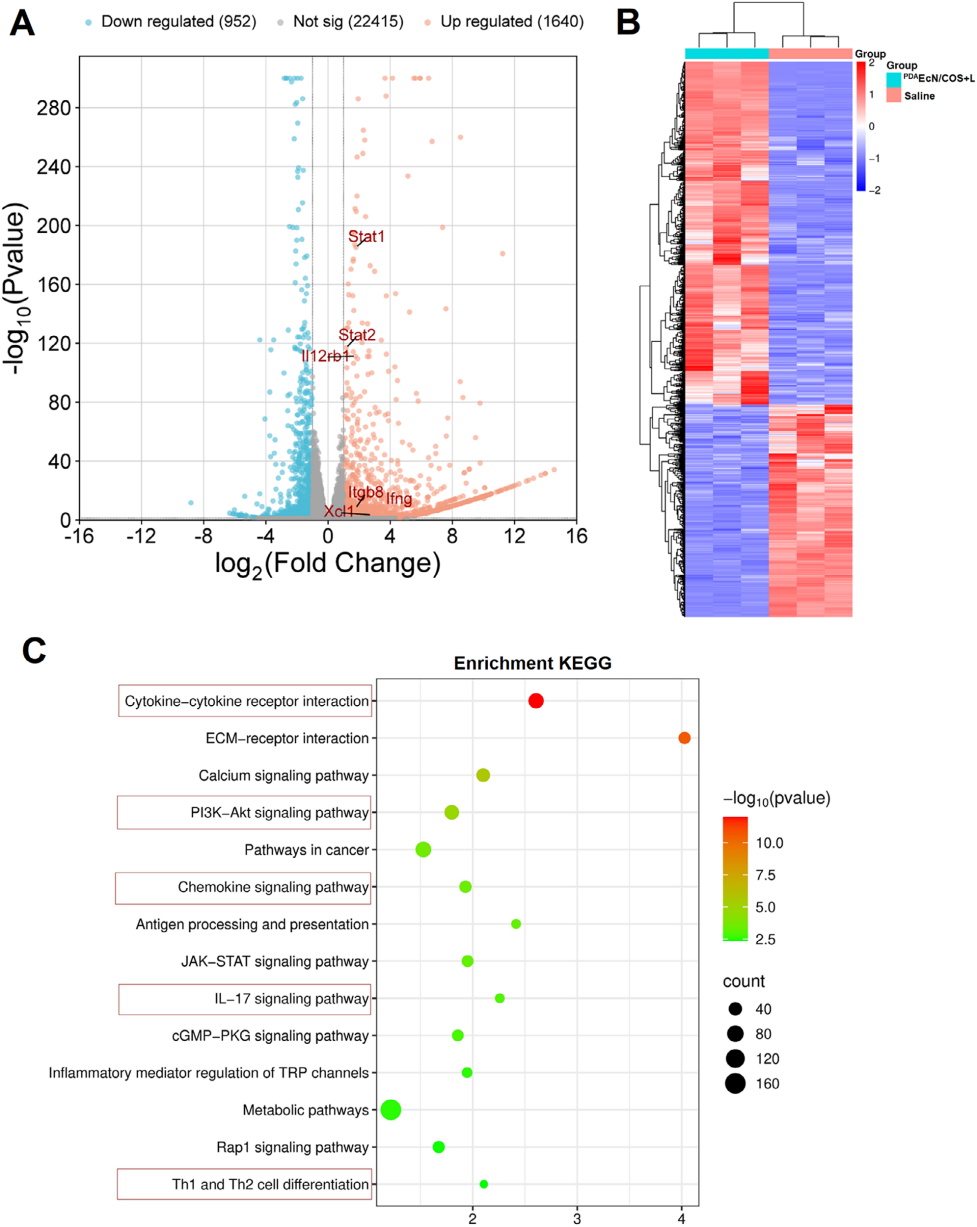
The underlying mechanism deduced from the transcriptome sequencing of whole 4T1 tumors after different treatments. Volcano plot showing the significantly upregulated (red dots) and downregulated (blue dots) mRNAs in 4T1 tumors A). Heatmap of the transcriptome sequencing results of whole tumors after different treatments B). KEGG analysis of differentially expressed mRNAs in 4T1 tumors after treatment with ^PDA^ECN/COS+L C).

Triple‐negative breast cancer (tumor model constructed by 4T1 cells) has typical characteristics of “cold” tumors, which are characterized by an overall immunosuppressive state in the tumor microenvironment, leading to limited functions of immune killer cells. Previous studies have shown that the immunogenic cell death (ICD) effect of mild PTT and chitosan oligosaccharides (COS) in the preparation can reverse immunosuppression by activating the tumor microenvironment and promoting immune cell infiltration. Based on this, we proposed to synergize the above‐mentioned immune activation mechanism with the tumor‐targeted enrichment of the bacterial system to enhance the anti‐tumor effect. To verify the immunomodulatory effect of ^PDA^ECN/COS+L, we performed transcriptome sequencing analysis on the tumor tissues (rather than the whole mice) of tumor‐bearing mice (n = 3) treated with ^PDA^ECN/COS+L and saline, aiming to systematically evaluate the reprogramming effect of this therapy on the tumor immune microenvironment through changes in gene expression profiles. As shown in **Figure** **10**A,B, we obtained a total of 2,592 differentially expressed genes, of which 1,640 were up‐regulated and 952 were down‐regulated. Most of the differentially expressed genes showed a trend related to immune activation after ^PDA^ECN/COS+L treatment. Subsequently, we performed pathway enrichment analysis on these genes. Surprisingly, we significantly enriched signaling pathways highly related to tumor immune activation, such as Cytokine‐cytokine receptor interaction, PI3K‐Akt signaling pathway, Chemokine signaling pathway, etc. (Figure 10C), which is consistent with our previous speculation.

### 2.10 In Vivo Antimetastatic Effects

**Figure 11:**
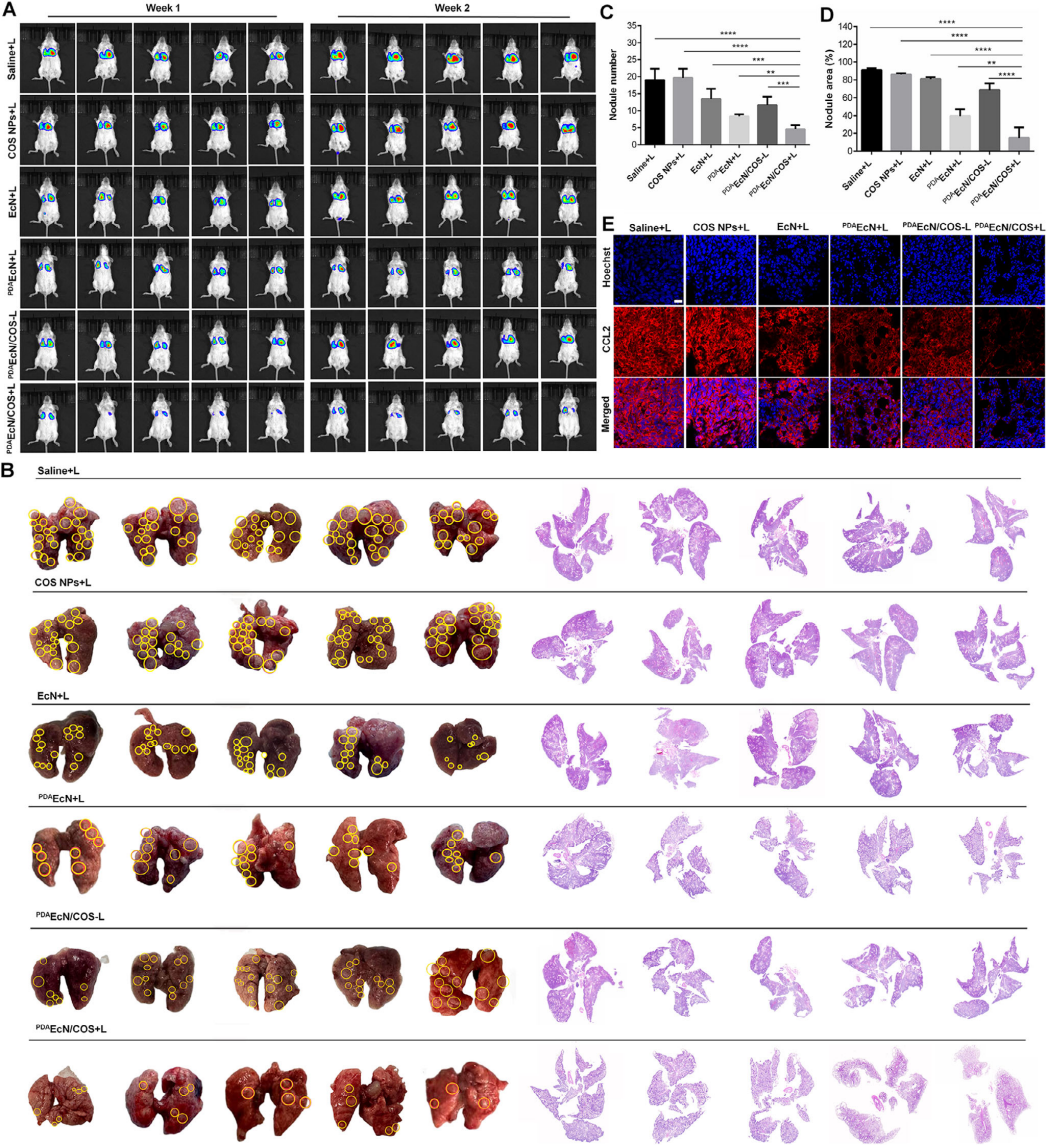
BLI imaging A), photos, and H&E staining B) for evaluating lung metastasis of mice after different treatments. Statistical results of lung metastatic nodules in photos C). Statistical results of lung metastatic nodule area based on H&E staining analysis D). IF images of CCL2 in the lung slices. Scale bar = 50 µm E). The mean values and S.D. were represented (n = 5). ^**^p < 0.01, ^***^p < 0.001, and ^****^p < 0.0001, and ns represents no significant difference.

Considering the highly aggressive behavior of 4T1 cancer cells, the potential anti‐metastatic effect of ^PDA^EcN/COS under irradiation in pulmonary tissues was investigated using the 4T1‐luciferase (4T1‐luc) breast cancer metastasis model. BALB/c mice were intravenously injected with 4T1^luc^ cells and subsequently randomized into six groups (n = 5 per group), followed by administration of saline+L, COS NPs+L, EcN+L, ^PDA^EcN+L, ^PDA^EcN/COS‐L, or ^PDA^EcN/COS+L, respectively. The development of metastatic nodules was monitored over two weeks via bioluminescence imaging. As illustrated in **Figure** **11**A, the most intense fluorescence signals in the lungs were observed in mice treated with saline+L, whereas markedly weaker fluorescence was recorded in the ^PDA^EcN/COS+L group, implying a potent inhibition of pulmonary metastasis by ^PDA^EcN/COS+L. Furthermore, photographs and H&E‐stained sections of lung tissues (Figure 11B) were employed to observe metastatic nodules. The findings revealed that lungs from saline+L‐treated mice exhibited a substantial number of metastatic lesions, whereas few nodules were discernible in the lungs of ^PDA^EcN/COS+L‐treated mice (Figure 11C,D). This outcome was attributed to the systemic immune activation induced by ^PDA^EcN/COS+L, which markedly suppressed lung metastasis of 4T1 tumors.

Macrophage infiltration within breast cancer tumors has been closely associated with tumor progression, metastatic spread, and poor clinical prognosis. CCL2 has been identified as a pivotal mediator facilitating TAM accumulation and promoting breast cancer metastasis to pulmonary and cerebral sites rather than to osseous tissues. Consequently, the expression of CCL2 in murine lungs was examined, revealing a significant reduction in the ^PDA^EcN/COS+L‐treated group compared with other treatment groups, as demonstrated by IF analysis (Figure 11E). It has been previously reported that the recruitment of inflammatory monocytes via CCR2‐the receptor for the chemokine CCL2‐and the subsequent accumulation of metastasis‐associated macrophages, alongside their interactions with metastatic tumor cells, are dependent upon CCL2 synthesized within the tumor and stromal compartments. Treatment with ^PDA^EcN/COS+L substantially diminished the expression levels of CCL2 and inhibited the metastatic potential of 4T1 cells to the lungs.

## 3 Conclusion 

In this study, we constructed an “all‐in‐one” extracellular anaerobic bacterial nanocomposite system, ^PDA^EcN/COS, to achieve target enrichment, self‐blocking of immune checkpoints, tumor‐associated macrophage polarization, and ICD elicited by mild PTT. The results indicated that ^PDA^EcN/COS could effectively eliminate tumors without relapse or metastasis with only one injection under laser irradiation. This strategy integrated bacterial therapy, immunotherapy, and photothermal effect using bacteria as the carrier, demonstrating excellent cancer treatment efficacy with minimal side effects.

## 4 Experimental Section

### Materials

Detailed information regarding the materials utilized in this experimental study is presented in the Supporting Information.

### Methods–Preparation and Characterization of ^PDA^EcN/COS–Preparation and Characterization of ^PDA^EcN

A volume of 50 µL of bacterial suspension was introduced into 5 mL of dopamine solution at varying concentrations (200, 400, 600, 800, 1,000, 1,500, and 2,000 µg mL^−1^). After constant agitation at ambient conditions for 2 h, ^PDA^EcN was collected by centrifugation at 4,000 g for 20 min, subsequently resuspended in sterile saline, and maintained at 4 °C for immediate utilization.

Hydrodynamic diameters, zeta potentials, and UV–vis absorption of ^PDA^EcN synthesized at various dopamine concentrations were measured using a NanoBrook 90PlusPALS analyzer (USA) and a PERSEE TU‐1901 UV–vis spectrophotometer (China), respectively. Infrared thermography was applied to monitor the temperature variations of ^PDA^EcN following 5 min of irradiation under different PDA concentrations or laser power conditions. Morphological images of pure EcN and ^PDA^EcN synthesized at 600 µg mL^−1^ were obtained using a JEOL JEM1400 transmission electron microscope (Japan). The interactions between PDA and EcN were investigated employing Nicolet IS50 FTIR spectroscopy (USA).

### Methods–Preparation and Characterization of ^PDA^EcN/COS–Preparation and Characterization of COS NPs

The COS NPs suspension was generated through electrostatic interactions between cationic COS (NH_3_⁺) and anionic PAA (COO^−^). Specifically, 1 mL of PAA (1 mg mL^−1^) was gradually dripped into 5 mL of COS (2 mg mL^−1^) under ultrasonication for 10–15 min until a homogeneous COS NPs suspension was achieved. The morphology, particle size, and zeta potential of COS NPs were characterized by employing a JEOL JEM1400 transmission electron microscope (Japan) and a NanoBrook 90PlusPALS analyzer (USA), respectively. In addition, Rhodamine B (RHB) was encapsulated within COS NPs for fluorescent tracing purposes.

### Methods–Preparation and Characterization of ^PDA^EcN/COS–Preparation and Characterization of ^PDA^EcN/COS

^PDA^EcN/COS was synthesized by the gradual addition of COS NPs to ^PDA^EcN under continuous stirring for 1 h, succeeded by centrifugation at 4,000 g for 20 min. The morphology and zeta potential of ^PDA^EcN/COS were characterized using a JEOL JEM1400 transmission electron microscope (Japan) and a NanoBrook 90PlusPALS analyzer (USA), respectively. The binding between ^PDA^EcN and fluorescent COS NPs was visualized employing an Olympus BX53F2 Fluorescent Microscope (Japan).

The change in the viability of EcN following irradiation was assessed using the Live/Dead BacLight bacterial viability kit. Specifically, ^PDA^EcN/COS, ^PDA^EcN, and EcN were exposed to irradiation at a power density of 1 W cm^−^^2^ for 5 min. Bacteria without irradiation served as controls. After overnight cultivation, bacterial cells underwent staining using SYTO 9 and PI fluorescent dyes during a 30‐min period at 37 °C under dark conditions. The fluorescently labelled bacterial cells were then observed through an Olympus BX53F2 Fluorescent Microscope (Japan).

The stability of PDA coating under simulated physiological conditions was also investigated by measuring the UV–vis absorption of ^PDA^EcN using a NanoBrook 90PlusPALS analyzer (USA) at different time points (0 h, 2 h, 4 h, 8 h, 12 h, 24 h, and 48 h).

### Tumor Cell Surface Colonization, Camouflaging, and Multicellular Spheroids Penetration Ability

4T1 cells were employed as the model system. Bacterial colonization on the surface of tumor cells was visualized utilizing scanning electron microscopy (SEM) and confocal laser scanning microscopy (CLSM). For SEM analysis, EcN, ^PDA^EcN and ^PDA^EcN/COS were co‐cultured with 4T1 cells for 2 h, respectively. Afterward, the 4T1 cells underwent collection, were subjected to fixation using 2.5% glutaraldehyde throughout the night, and experienced dehydration via an ethanol concentration series. The specimens were then thoroughly dried and sputter‐coated with gold before SEM (Zeiss EVO LS15, Oberkochen, Germany) imaging. For CLSM visualization, green fluorescent EcN^GFP^, ^PDA^EcN^GFP^ and ^PDA^EcN^GFP^/COS were introduced into 4T1 cells and incubated for 2 h, respectively. Following staining with Hoechst 33258, bacterial colonization on the tumor surface was observed using CLSM (Zeiss LSM 880, Germany).

The self‐blocking capabilities of ^PDA^EcN/COS were examined. Specifically, PBS, EcN, ^PDA^EcN, and ^PDA^EcN/COS were each co‐incubated with 4T1 cells for 2 h. The samples underwent fixation using 4% paraformaldehyde solution for 15 min. Thereafter, all cells were permeabilized with 0.1% Triton‐X solution and blocked utilizing 10% goat serum solution. Following this, the cells were incubated with PD‐L1 and CD47 antibodies at 4 °C overnight. Lastly, the nuclei underwent staining with Hoechst 33258, and subsequent observation of the samples occurred through CLSM (Zeiss LSM 880, Germany).

PD‐L1 and CD47 levels were further analyzed using western blotting. Specifically, 4T1 cells were incubated with PBS, EcN, ^PDA^EcN, and ^PDA^EcN/COS for 2 h, respectively. Subsequently, the treated cells were collected and subjected to lysis at 4 °C for 30 min using RIPA lysis buffer containing protease inhibitor cocktail. Following centrifugation at 12 000 r min^−1^ for 15 min, the supernatant was obtained, and protein content was measured utilizing the BCA protein assay kit. The protein specimens were then resolved by sodium dodecyl sulfate‐polyacrylamide gel electrophoresis and transferred to polyvinylidene fluoride membranes. The membranes were subsequently incubated with primary antibodies targeting PD‐L1 or CD47 overnight at 4 °C, succeeded by treatment with appropriate secondary antibodies for 1 h at ambient conditions. Finally, the membranes were visualized using an ECL imaging system.

To evaluate the multicellular spheroids penetration of ^PDA^EcN/COS, red fluorescent 4T1^RFP^ cells in the logarithmic growth phase were placed into 96‐well ultra‐low adhesion plates at 4 × 10⁴/well and centrifuged at 800 g for 10 min at ambient conditions. The plates were then incubated, with the culture medium replaced every other day over 15 days, during which the formation of regular multicellular spheroids was monitored using microscopy. On day 15, EcN^GFP^, ^PDA^EcN^GFP^ and ^PDA^EcN^GFP^/COS were introduced and maintained with the multicellular spheroids for 3 h. Subsequently, the multicellular spheroids were washed with PBS buffer and stabilized using 4% paraformaldehyde for 15 min. CLSM (Zeiss LSM 880, Germany) scanning along the Z‐axis was performed at 1 µm intervals to visualize the distribution of fluorescent bacteria within the multicellular spheroids.

### Mild PTT In Vitro–Live/Dead Assay

4T1 cells were treated with PBS, COS NPs, EcN, ^PDA^EcN, or ^PDA^EcN/COS with or without irradiation. For the irradiation groups, exposure to irradiation occurred after 2 h of co‐incubation at a power density of 1 W cm^−^^2^ for 5 min, succeeded by a further incubation period of 12 h. Subsequently, the cells were stained using a calcein‐AM/PI live/dead staining assay kit. Finally, the fluorescence of 4T1 cells was visualized by CLSM (Zeiss LSM 880, Germany).

### Mild PTT In Vitro–In Vitro ICD Response Assessment

Typically, 4T1 cells were initially placed into 24‐well plates at 1 × 10⁵ cells per well. Following 24 h of incubation, the cells were exposed to a fresh medium containing PBS+L, COS NPs+L, EcN+L, ^PDA^EcN+L, ^PDA^EcN/COS‐L, or ^PDA^EcN/COS+L for 2 h. The treatment proceeded with illumination at 1 W cm^−^^2^ density for 5 min, succeeded after a 12 h incubation period. Immunofluorescence staining was employed to evaluate the intracellular high mobility group box 1 (HMGB1) content in 4T1 cells exposed to various treatments. The protocol involved fixing the cells using 4% paraformaldehyde, followed by washing and membrane permeabilization with 0.1% Triton X‐100 for 10 min. The specimens were then subjected to blocking with 10% normal goat serum (NGS) before overnight exposure to anti‐HMGB1 antibody at 4 °C. Post‐treatment with Alexa Fluor 488 secondary antibodies lasting 1 h, the specimens underwent counterstaining with Hoechst 33258. The final observation was conducted through CLSM (Zeiss LSM 880, Germany).

### Mild PTT In Vitro–ICD‐Induced DC Maturation

To examine the DC maturation process in vitro, DC2.4 cells were cultured in PRMI‐1640 medium. Immature DCs and 4T1 cells were co‐cultured overnight using a Transwell system. Subsequently, the tested agents, including PBS+L, COS NPs+L, EcN+L, ^PDA^EcN+L, ^PDA^EcN/COS‐L, and ^PDA^EcN/COS+L, were co‐incubated with 4T1 cells for 12 h. Irradiation was applied after 2 h of incubation at 1 W cm^−^^2^ lasting 5 min. Following staining with anti‐CD80‐PE and anti‐CD86‐APC, DC maturation status was assessed using BD FACSCanto II flow cytometry (FCM, USA). Meanwhile, TNF‐α and IL‐6 levels present in the supernatant samples were quantified utilizing ELISA kits.

### Mild PTT In Vitro–Cell Viability Assay

To validate the enhanced tumor‐killing effect mediated by T cells, in vitro cytotoxicity assays were conducted. Initially, 4T1 cells (2 × 10⁴ cells per well) were seeded into 96‐well microplates. Following a 4 h incubation, spleen cells (4 × 10⁵ cells per well), isolated from 4T1‐bearing BALB/c mice, were introduced together with PBS+L, COS NPs+L, EcN+L, ^PDA^EcN+L, ^PDA^EcN/COS‐L, or ^PDA^EcN/COS+L and co‐incubated for a further 12 h. Irradiation was applied after 2 h of incubation at a power density of 1 W cm^−^^2^. After incubation, the cultures were rinsed and subsequently exposed to 100 µL of 10% MTT within serum‐free medium for 4 h. Subsequently, 100 µL of DMSO was added, and incubation continued for an additional 10 min under oscillation. Ultimately, a versatile microplate reader instrument was utilized to determine the absorbance values of individual wells at 490 nm. Cell viability was computed according to the following equation:

(1) $$\mathrm{Cell}\;\mathrm{viability}\;\;\left(\%\right)=\frac{A_{sample\;}-A_{blank\;}}{A_{negative\;control\;}-A_{blank\;}}\times 100\%$$

In this context, for groups without T cells, A_negative control_ control referred to the absorbance of untreated 4T1 cells; for groups containing T cells, A_negative control_ control represented the absorbance of untreated 4T1 cells co‐cultured with spleen cells. A_blank_ corresponded to the absorbance of wells in the absence of cells.

### Macrophage Activation and Phagocytosis–Macrophage Polarization

Macrophages were placed into 6‐well plates with 5 × 10^5^ cells in each well. After IL‐4 stimulation for 24 h, the culture medium was exchanged with a new medium comprising PBS+L, COS NPs+L, EcN+L, ^PDA^EcN+L, ^PDA^EcN/COS‐L, or ^PDA^EcN/COS+L. For the irradiation groups, exposure to irradiation was conducted after 2 h of incubation (808 nm, 1 W cm^−^^2^, 5 min). After a further 24 h of culture, all cells were harvested by centrifugation. Anti‐CD80 (PE) and anti‐F4/80 (APC) antibodies were introduced and maintained at the recommended concentration for 30 min in the dark, succeeded by centrifugation to remove unbound antibodies and resuspension in PBS for FCM analysis. The detection of CD206 was performed similarly to CD80 after treatment with permeabilization wash buffer. Supernatants were collected by centrifugation and analyzed using TNF‐α and IL‐6 assay kits.

### Macrophage Activation and Phagocytosis–Macrophage Phagocytosis Assay

4T1^RFP^ cells were plated into 6‐well plates at 1 × 10⁵ cells per well and maintained overnight at 37 °C. Subsequently, 5 × 10⁶ green fluorescent RAW264.7^GFP^ cells, treated with PBS+L, COS NPs+L, EcN+L, ^PDA^EcN+L, ^PDA^EcN/COS‐L, and ^PDA^EcN/COS+L, were added and co‐cultured for an additional 1 and 6 h. Irradiation was applied at a power density of 1 W cm^−^^2^ for 5 min. CLSM (Zeiss LSM 880, Germany) was employed to visualize the phagocytosis of tumor cells by macrophages.

### Macrophage Activation and Phagocytosis–Cell Viability Assay

A similar experimental procedure was performed following “Section 2.2.3.4”, except that macrophages or a mixture of macrophages and T cells were used in place of T cells.

### Bioactivity and Cytotoxic Potential of ^PDA^EcN/COS

The bioactivity and cytotoxic potential of ^PDA^EcN/COS were assessed by quantifying the production of various cytokines. Specifically, female BALB/c mice received intravenous administration of 100 µL solutions containing COS NPs+L, EcN+L, ^PDA^EcN+L, ^PDA^EcN/COS‐L, or ^PDA^EcN/COS+L, respectively. Saline was administered as the control. Blood specimens were obtained at specified intervals (days 0, 1, 4, 7, and 10) followed by biochemical assessment and routine blood analysis.

The long‐term safety of ^PDA^EcN/COS was evaluated. Female BALB/c mice were repeatedly injected with 100 µL of COS NPs+L, EcN+L, ^PDA^EcN+L, ^PDA^EcN/COS‐L, or ^PDA^EcN/COS+L on days 0, 3, and 6, respectively. Saline was administered as the control. On day 21, the mice were sacrificed. Murine blood samples were collected and subsequently analyzed using a biochemistry assay and a blood routine analyzer, while organs were subjected to H&E staining for toxicity evaluation.

The LPS expression of EcN, ^PDA^EcN, or ^PDA^EcN/COS was investigated. Specifically, paraformaldehyde (4%) was applied to fix the cells for 30 min. Subsequently, cell permeabilization occurred using a 0.1% Triton‐X solution, while blocking was performed with a 10% NGS solution. Thereafter, the samples underwent overnight incubation at 4 °C with the LPS antibody, followed by exposure to a Dylight 649 secondary antibody for 1 h. The final step involved staining the cells with Hoechst 33258 and observation through CLSM (Zeiss LSM 880, Germany).

### In Vivo Biodistribution and Photothermal Effect

Tumors were established by inoculating 100 µL of 4T1 cells at a concentration of 3 × 10⁷ cells into the right flank of each female BALB/c mouse (6 weeks old, 18–20 g) under anesthesia. Once the tumor volume reached ≈150 mm^3^, 100 µL of EcN^GFP^, ^PDA^EcN^GFP^, or ^PDA^EcN^GFP^/COS were administered via tail vein injection. This time point was designated as day 0. Mice were sacrificed on days 1, 3, and 7 following injection, and their hearts, livers, spleens, lungs, kidneys, and tumors were collected and analyzed using an IVIS® Spectrum imaging system (PerkinElmer, USA). Furthermore, bacterial colony‐forming units (CFUs) within organs and tumors were quantified through tissue homogenization in sterile H_2_O comprising 0.1% Triton X‐100, subsequently plating sequential dilutions of the homogenized samples onto solid Luria‐Bertani (LB) agar plates and enumerating colonies following a 24‐h incubation at 37 °C.

For the photothermal effect assay, tumor‐bearing female BALB/c mice were injected with 100 µL of saline or ^PDA^EcN/COS via the tail vein. Irradiation was performed on day 4 post‐injection at a power density of 1 W cm^−^^2^ for 5 min. Thermal images of the entire mouse were captured using an infrared thermal camera, with images recorded at 1 min intervals following irradiation.

### In Vivo Antitumor Efficiency of ^PDA^EcN/COS

4T1 tumor‐bearing female BALB/c mice (6 weeks old, ≈20 g) were established as previously described. When tumor volumes approached 80 mm^3^, 100 µL of saline+L, COS NPs+L, EcN+L, ^PDA^EcN+L, ^PDA^EcN/COS‐L, or ^PDA^EcN/COS+L (10⁶ CFU mL^−1^) was administered via tail vein injection. The initial moment was marked as day 1. By day 3, the experimental mice underwent irradiation treatment for 5 min utilizing a near‐infrared laser (808 nm, 1 W cm^−^^2^). Throughout a 22‐day period, measurements of tumor size, body mass, and survival rates were recorded at 2‐day intervals. Upon reaching day 22, the animals were euthanized, succeeded by the extraction of spleens and tumors. Tumor slices were stained with H&E to evaluate tumor necrosis, while immunohistochemical staining of Ki‐67 and caspase‐3 was performed to assess tumor cell proliferation and apoptosis.

### Immune Activation of ^PDA^EcN/COS In Vivo

To evaluate the immunological effects triggered by the combined treatment, tumors and spleens were extracted from mice across various experimental groups. The tumor and spleen tissues were subsequently excised and digested at 37 °C for 1 h. Thereafter, the cellular preparation was passed through a 70 µm filtration membrane to generate an individual‐cell suspension. For spleen cells, red blood cell lysate (3 mL) was then added to remove erythrocytes. Next, different concentrations of percoll were used to isolate the desired cells. Following this, 1 × 10⁵ cells were transferred into each FCM tube, and immune cells were stained for analysis (T cells: anti‐CD45‐FITC, anti‐CD4‐PE, anti‐CD8a‐PerCP‐Cy5.5; DC maturation: anti‐CD11c‐Pacific Blue A, anti‐CD80‐PE, anti‐CD86‐APC; Treg: anti‐CD45‐FITC, anti‐CD4‐PE, anti‐Foxp3‐APC; M1 and M2 macrophages: anti‐F4/80‐APC, anti‐CD80‐PE, anti‐CD206‐FITC).

For immunofluorescence (IF) staining, tumor slices were first blocked with 3% bovine serum albumin and subsequently incubated overnight at 4 °C with primary antibodies (anti‐HMGB1, anti‐PD‐L1, anti‐CD47, anti‐CD8, anti‐CD4, anti‐CD80, and anti‐CD206). Following this, the sections were stained with appropriate conjugated secondary antibodies for 1 h and then incubated with the corresponding TSA for 10 min. Finally, cell nuclei were stained with DAPI for 10 min. Fluorescence images were captured using a fluorescence microscope.

### Transcriptome Sequencing Tumor Tissues

Six female BALB/c mice were arbitrarily split into two groups (n = 3 per group): saline and ^PDA^EcN/COS+L. Each mouse received a 100 µL subcutaneous injection of 4T1 cell suspension (3 × 10⁷ cells mL^−1^) into the flank region. Once the average tumor volume reached 100 mm^3^ (designated as day 0), intravenous injections of saline, or ^PDA^EcN/COS were administered via the tail vein. On day 3, mice assigned to the irradiation groups underwent near‐infrared (NIR) laser treatment (808 nm, 1 W cm^−^^2^) for 5 min. On day 14, all mice were euthanized. Tumors were excised and fixed in 4% paraformaldehyde. RNA was subsequently extracted from each tumor, and RNA sequencing was executed. Differential gene expression analysis was executed to assess trends in target genomic expression.

### Lung Metastasis Assay

Initially, 100 µL of 4T1^luc^ cells (3 × 10⁷/mL) were intravenously injected into the mice. After one week, tail vein injections of saline+L, COS NPs+L, EcN+L, ^PDA^EcN+L, ^PDA^EcN/COS‐L, or ^PDA^EcN/COS+L (10⁶ CFU mL^−1^) were administered. Irradiation was performed three days post‐injection at a power density of 1 W cm^−^^2^ for 5 min. Lung metastasis was subsequently monitored using an IVIS® Spectrum imaging system (PerkinElmer, USA) at weeks 1 and 2. Following two weeks, the mice were euthanized, lungs were sectioned, and slices were stained with H&E to observe lung metastatic nodules. IF staining of the lung sections was conducted to examine the expression of chemokine ligand 2 (CCL2).

### Statistical Analyses

Data were presented as mean ± standard deviation (S.D.). Comparisons between the two groups were performed using the Student's *t*‐test. Statistical significance was indicated as follows: ^*^ *p* < 0.05, ^**^ *p* < 0.01, ^***^ *p* < 0.001, ^****^ *p* < 0.0001, and ns represents no significant difference.

### Ethics Approval and Consent to Participate

All procedures involving animals were conducted in compliance with the Guidelines for the Care and Use of Laboratory Animals at Binzhou Medical University and were approved by the Animal Ethics Committee of Binzhou Medical University (Approval No. 2023–410).

## Conflict of Interest

The authors declare no conflict of interest.

## Consent for publication

All authors agree to submit for publication in “Advanced Science”.

## Availability of data and materials

No datasets were generated or analyzed during the current study.

## Supporting information



Supporting Information

## Data Availability

Data available on request due to privacy/ethical restrictions
