# Supplementary material for: Targeted Enrichment of Engineered Bacteria on Triple‐Negative Breast Cancer Turns Immunologically Cold Tumors Hot
Source: Adv Sci (Weinh). 2025 Sep 30;12(47):e10171. doi: 10.1002/advs.202510171 (PMC12713098; doi:10.1002/advs.202510171)
Supplement: Supplementary file 1 — Supporting Information [file ADVS-12-e10171-s001.docx]

***Supporting Information***

**Targeted Enrichment of Engineered Bacteria on Triple-Negative Breast Cancer Turns Immunologically Cold Tumors Hot**

Xuanxiang Zhai^1^, Xiaoyi Shi^1^, Xiao Liu, Xiaotong Gu, Wenting Li*, Xiangjun Chen*, Wei Hong*

*School of Pharmacy, Shandong New Drug Loading & Release Technology and Preparation Engineering Laboratory, Binzhou Medical University,* *346 Guanhai Road, Yantai, 264003, P. R. China*

^*^*Corresponding authors.* H.W. *Tel./Fax: +86-**535-6908191,*

*E-mail address: hongwei_sy@bzmc.edu.cn*

C. XJ. *Tel./Fax: +86-535-6908191,*

*E-mail address:* *chenxj@bzmc.edu.cn*

L.WT. *Tel./Fax: +86-535- 6913719,*

*E-mail address: liwenting1003@bzmc.edu.cn*

Xuanxiang Zhai and Xiaoyi Shi contributed equally to this work.

**1. Materials**

**1.1 Reagents**

Dopamine hydrochloride was purchased from Anhui Zesheng Technology Co., Ltd (Anhui, China). Chitosan oligosaccharide was purchased from Heowns Biochemical Technology Co., Ltd (Tianjin, China). PD-L1/CD274 Mouse McAb and MCP-1/CCL2 Rabbit PolyAb were purchased from Proteintech group (Wuhan, China). Anti-Mo CD11c, Anti-Mo CD45, Anti-Mo CD8a, Anti-Mo CD80, Anti-Mo CD4, Anti-Mo F4/80, Anti-Mo CD206, Anti-Mo CD86 and Anti-Mo/Rt FOXP3 were purchased from Thermo Fisher Scientific(USA). A LIVE/DEAD^®^ BacLight™ Bacterial Viability Kit (L7012) was purchased from Thermo Fisher Scientific Inc (Shanghai, China). Fetal bovine serum and penicillin/streptomycin mixture was obtained from Wuhan Punosai Life Sciences Co., Ltd (Wuhan, Hubei, China). 4% paraformaldehyde was obtained from LABGIC Co., Ltd (Beijing, China). Thiazolyl blue and Percoll was obtained from Solarbio Biotechnology Co., Ltd (Beijing, China).

**1.2 Bacteria and cells**

*Escherichia coli* Nissle1917 (EcN) and *Escherichia coli* Nissle1917^GFP^ (EcN^GFP^) were purchased from BeNa Culture Collection (Henan, China). 4T1 cells (RRID: CVCL-0125), DC2.4 cells (RRID: CVCL-J409), and RAW264.7 cells (RRID: CVCL-0493) were purchased from BeNa Culture Collection (Henan, China), and cultured in basic culture medium containing 10% FBS. 4T1^RFP^ cells, 4T1^luc^ cells and RAW264.7^GFP^ cells were constructed by BeNa Culture Collection (Henan, China) basing on 4T1 cells (RRID: CVCL-0125) and RAW264.7 cells (RRID: CVCL-0493), respectively. All of the cell lines were contamination free.

**1.3 Animals**

Female BALB/c mice were purchased from Pengyue Laboratory Animal Breeding Co., Ltd (Jinan, Shandong, China). The animal studies were conducted according to the experimental protocols by Institutional Animal Care and Use Committee of Binzhou Medical University (Approval No. 2023-410).

**2. Results**


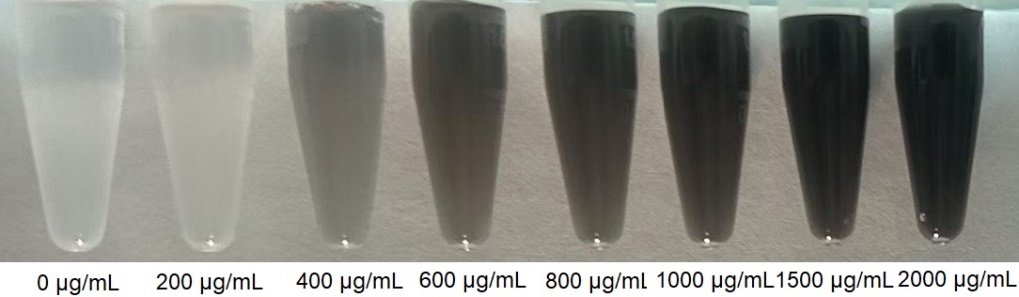


**Fig. 1** The photos of ^PDA^EcN prepared at 0-2000 μg/mL of dopamine.


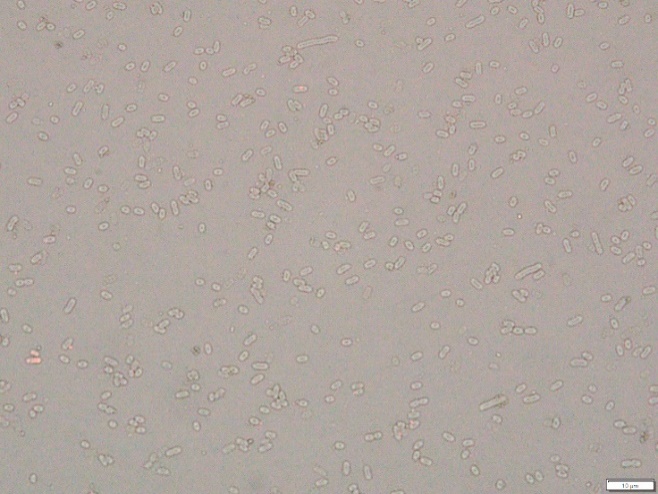


**Fig. 2** The fluorescent image of EcN/COS@RHB. Scale bar=10 μm.


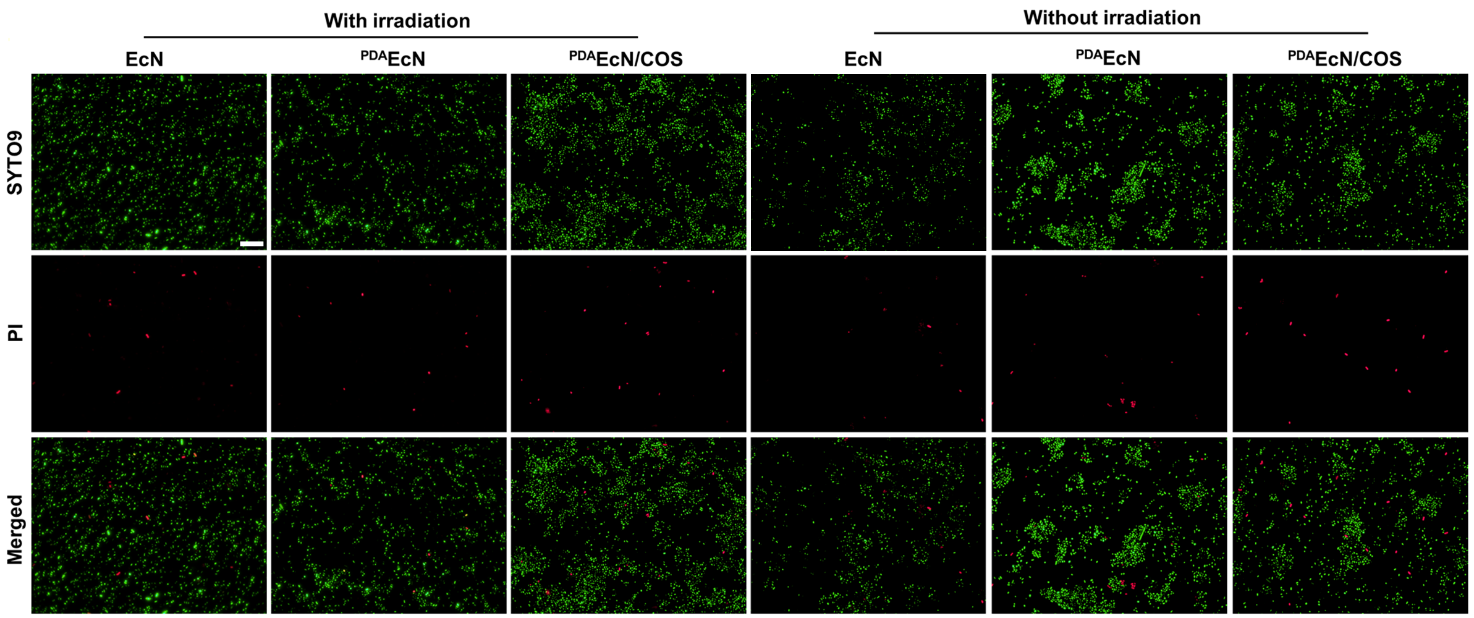


**Fig. 3** Live/Dead assay of EcN, ^PDA^EcN and ^PDA^EcN/COS with and without irradiation. Scale bar=20 μm.





**Fig. 4** Bacteria suspension concentrations of EcN, ^PDA^EcN and ^PDA^EcN/COS determined by viable count on solid LB agar plated every 2 h.





**Fig. 5** The stability of PDA coating of ^PDA^EcN under simulated physiological condition.


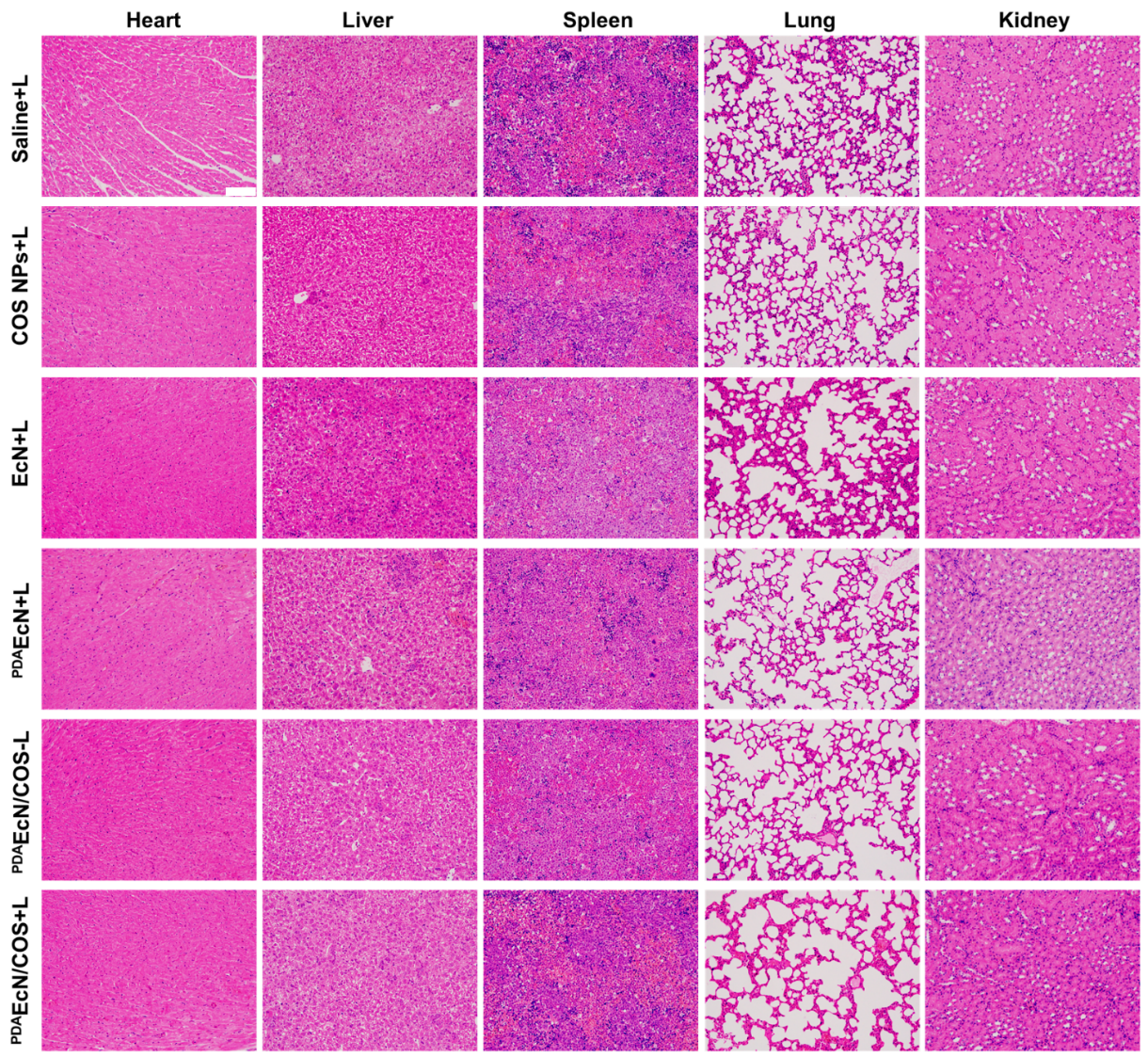


**Fig. 6** The H&E staining of major organs after treatment with different agents in female BALB/c mice (n = 5). Scale bar = 50 μm.


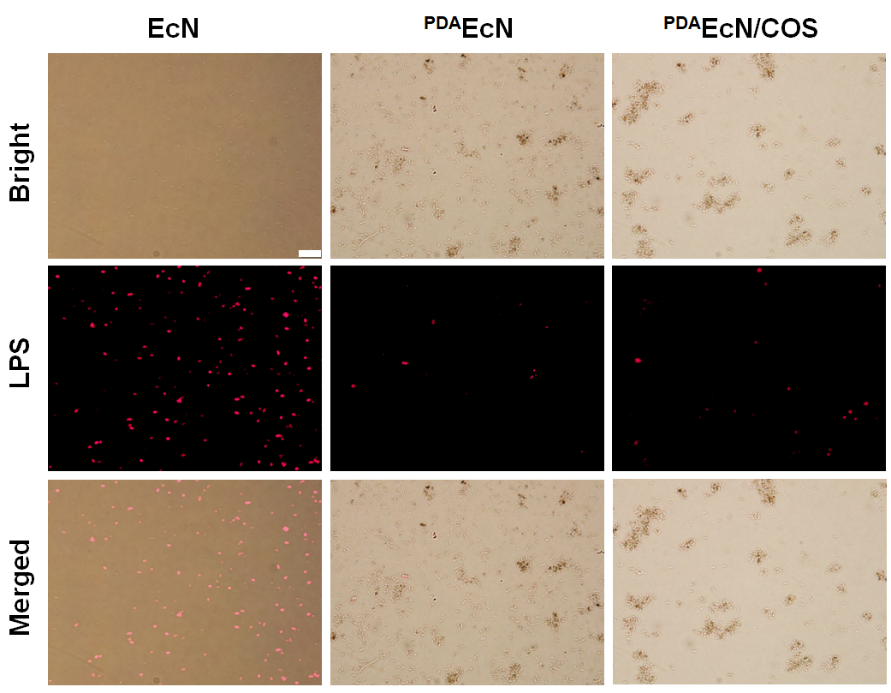


**Fig. 7** The LPS expression of EcN, ^PDA^EcN, and ^PDA^EcN/COS. Scale bar=10 μm.


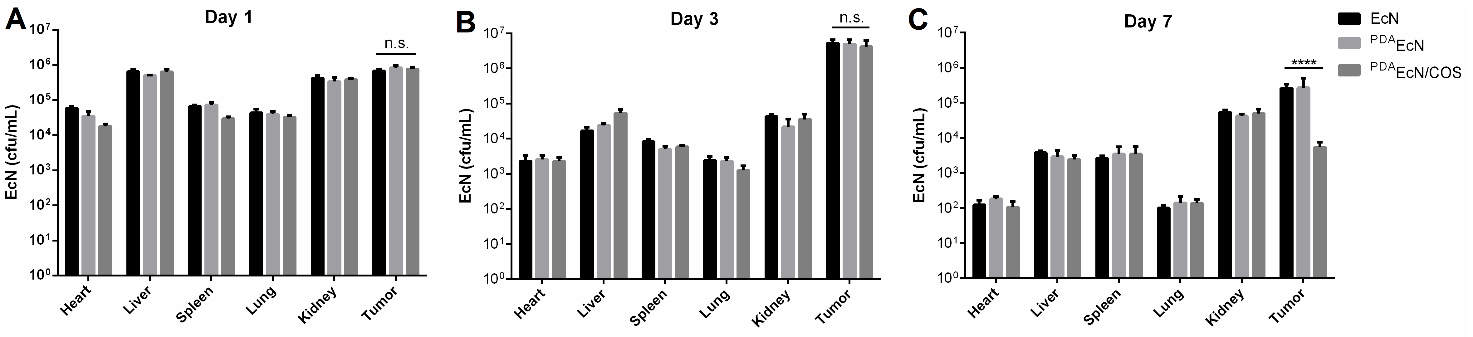


**Fig. 8** Bacterial numbers of EcN^GFP^, ^PDA^EcN^GFP^ and ^PDA^EcN^GFP^/COS in tumors and in heart, liver, spleen, lung and kidney on day 1 (A), day 3 (B) and day 7 (C). Data are presented as mean ± SEM, n=3, *****p* < 0.0001, and ns represents no signiﬁcant diﬀerence.


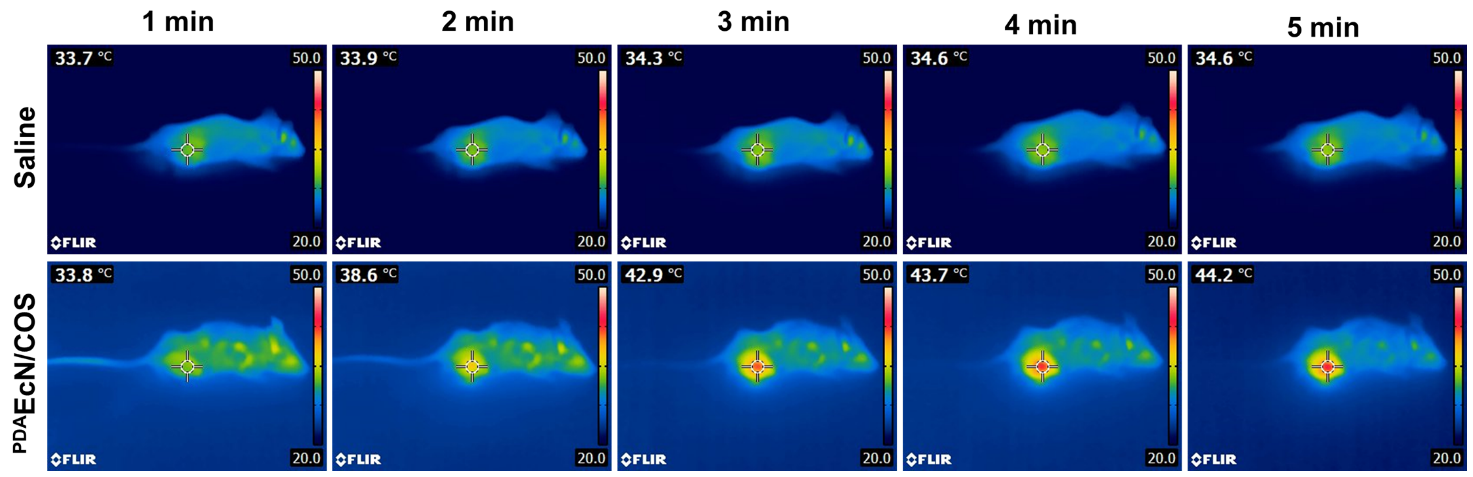


**Fig. 9** Thermal images of tumor-bearing female BALB/c mice injected with saline or ^PDA^EcN/COS (100 μL) *via* tail vein. Irradiation was performed on day 3 post-injection at a power density of 1 W/cm² for 5 min.


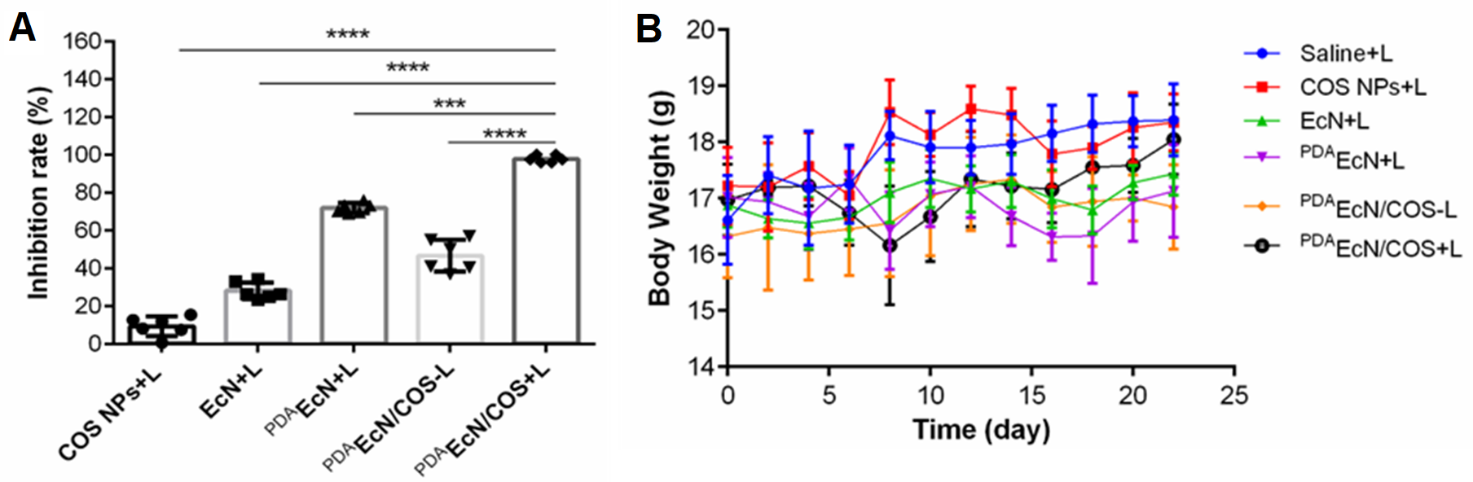


**Fig. 10** The inhibition rate of tumors in diﬀerent groups, relative to the tumor volume in the saline group. Average body weights of mice after receiving various treatments. Data are presented as mean ± SEM, n=6, ****p* < 0.001 and *****p* < 0.0001.
